# Using machine learning to develop a clinical prediction model for SSRI-associated bleeding: a feasibility study

**DOI:** 10.1186/s12911-023-02206-3

**Published:** 2023-06-11

**Authors:** Jatin Goyal, Ding Quan Ng, Kevin Zhang, Alexandre Chan, Joyce Lee, Kai Zheng, Keri Hurley-Kim, Lee Nguyen, Lu He, Megan Nguyen, Sarah McBane, Wei Li, Christine Luu Cadiz

**Affiliations:** 1 Donald Bren School of Information and Computer Sciences, University of California Irvine, Irvine, CA USA; 2 Department of Clinical Pharmacy Practice, School of Pharmacy and Pharmaceutical Sciences, University of California Irvine, 802 W Peltason Dr, Irvine, CA 92697-4625 USA; 3 Division of Computational Biomedicine, Department of Biological Chemistry, School of Medicine, University of California Irvine, Irvine, CA USA

**Keywords:** Machine learning, Adverse drug events, Selective serotonin-reuptake inhibitors, Bleeding, Electronic health records

## Abstract

**Introduction:**

Adverse drug events (ADEs) are associated with poor outcomes and increased costs but may be prevented with prediction tools. With the National Institute of Health *All of Us (AoU)* database, we employed machine learning (ML) to predict selective serotonin reuptake inhibitor (SSRI)-associated bleeding.

**Methods:**

The *AoU* program, beginning in 05/2018, continues to recruit ≥ 18 years old individuals across the United States. Participants completed surveys and consented to contribute electronic health record (EHR) for research. Using the EHR, we determined participants who were exposed to SSRIs (citalopram, escitalopram, fluoxetine, fluvoxamine, paroxetine, sertraline, vortioxetine). Features (*n* = 88) were selected with clinicians’ input and comprised sociodemographic, lifestyle, comorbidities, and medication use information. We identified bleeding events with validated EHR algorithms and applied logistic regression, decision tree, random forest, and extreme gradient boost to predict bleeding during SSRI exposure. We assessed model performance with area under the receiver operating characteristic curve statistic (AUC) and defined clinically significant features as resulting in > 0.01 decline in AUC after removal from the model, in three of four ML models.

**Results:**

There were 10,362 participants exposed to SSRIs, with 9.6% experiencing a bleeding event during SSRI exposure. For each SSRI, performance across all four ML models was relatively consistent. AUCs from the best models ranged 0.632–0.698. Clinically significant features included health literacy for escitalopram, and bleeding history and socioeconomic status for all SSRIs.

**Conclusions:**

We demonstrated feasibility of predicting ADEs using ML. Incorporating genomic features and drug interactions with deep learning models may improve ADE prediction.

**Supplementary Information:**

The online version contains supplementary material available at 10.1186/s12911-023-02206-3.

## Key points

We used machine learning and found bleeding history and socioeconomic status are important for predicting SSRI-related bleeding. Neural networks with genomic features are planned for future analyses.

## Introduction

The advent of modern medicines has improved the lives of millions worldwide. In the United States (US), more than one billion medications are prescribed in a single year [1]. Medications are prescribed with the intent of improving patients’ lives, yet unintended adverse drug events (ADEs) may occur. ADEs cause approximately 1.3 million emergency department visits and 350,000 hospitalizations each year in the US [2]. These hospitalizations are often prolonged and may precipitate secondary health problems [3]. The Agency for Healthcare Research and Quality reported an 11.3% increase in hospitalizations that involved an ADE present upon admission in the US between 2010 and 2014 [4]. The mean cost per hospital stay also increased by 15% for ADEs that were present on admission but doubled if they originated during the hospital stay [4].

Studies have shown that approximately 80% of ADEs are predictable, with more than 40% of ADE-attributable healthcare costs being preventable [5, 6]. The ability to predict and prevent ADEs in clinical practice would minimize harm and associated financial burden. Traditional efforts have focused mainly on system measures such as electronic prescribing and automated dispensing to minimize human error, but do not account for the underlying risk of ADEs for individual patients [7]. Precision medicine may play a key role in preventing ADEs through a holistic review of patients’ sociodemographic, clinical, and omics profiles to predict risk of future ADEs at time of prescribing or admission [8, 9].

A use case of precision medicine in ADE research is the prediction of bleeding events after exposure to selective serotonin reuptake inhibitors (SSRIs), a rare but debilitating side effect of SSRIs that can cause significant morbidity and hospitalizations [10, 11]. SSRIs are commonly prescribed to manage psychiatric conditions such as depressive and anxiety disorders across all ages [12], as well as off-label uses for conditions such as post-stroke recovery [13]. The pharmacologic properties of SSRIs stem from their effect of increasing serotoninergic activity at neuronal synapses [14]. However, off-target effects have been observed, including reductions in platelet serotonin content of 80–90% with sustained SSRI exposure [15–17]. Serotonin changes in the platelet microenvironment are postulated to explain the higher coronary artery events in depressed geriatric patients, antithrombotic effects of SSRIs, and increased bleeding risk with SSRI exposure [18, 19]. This is notwithstanding the multiplicative effect of SSRIs on bleeding through increasing gastric acid secretion and inhibiting cytochrome-P450 (CYP) enzymes [11, 19], as well as patient-level differences in CYP-enzyme genetic variants that explain interindividual pharmacokinetic differences and bleeding risks [20]. Therefore, in this study, we employed machine learning (ML) techniques to account for these complex relationships in the prediction of SSRI-associated bleeding events and leveraged the large datasets collected by the *All of Us* (*AoU) *Research Program for model development and validation [21].

## Methods

### Data source

The *AoU *program, a National Institutes of Health (NIH) initiative [22], aims to enhance healthcare through facilitating precision medicine research, recruiting one million plus participants nationwide, and providing researchers with access to participants’ electronic health records (EHR) and survey data to define clinical features and outcomes for prediction model development [23]. The *AoU *program began in May 2018 and continues to recruit individuals 18 years old or older across more than 340 recruitment sites around the US [23]. All data [electronic health records (EHR) and surveys] are organized with the Observational Health and Medicines Outcomes Partnership (OMOP) common data model v5.2 [24]. This study does not require Institutional Review Board approval as the authors were not involved in any direct interaction with participants and all data have been de-identified by the *AoU* research team. All researchers must adhere to the *AoU* Data User Code of Conduct for upholding data privacy and confidentiality.

### Study design and sample

Participants who received clopidogrel, warfarin and SSRIs (citalopram, escitalopram, fluoxetine, fluvoxamine, paroxetine, sertraline and vortioxetine) were identified with the EHR. Clopidogrel and warfarin were analyzed concurrently with SSRIs to serve as positive controls. The OMOP concept identifications (IDs) for identifying exposure to these drugs are listed in eTable 1 of the Supplement. We created a total of nine individual drug cohorts and one combined SSRI cohort comprising all patients receiving different types of SSRIs. Each cohort of participants were used to create independent prediction models for the respective medications (individual SSRIs, all SSRIs combined, clopidogrel, and warfarin). To ensure adequacy of EHR data for analysis, eligible patients must have at least one recorded visit to the EHR institution during the 365 days before the index date, and one record of visit during the follow-up period.

1. Index date: The index date, also known as cohort entry date, is the first drug exposure date of each medication for the respective drug cohorts. The index date was identified using dispensing and administration records. To reduce the risk of immortal time bias, prescription records were not used to define index dates.
2. Follow-up period: The follow-up period was defined by continuous records of dispensing, administration, and prescription of the medications of interest. Follow-up of patients continued until the occurrence of bleeding event or if there was lack of evidence of medication exposure for ≥ 90 days. For the combined SSRI cohort, SSRI switching served as an additional criterion for determining follow-up end date. Cohort re-entry was permitted.

### Bleeding event outcome algorithm

Bleeding events were identified during the follow-up period. All healthcare data were stored using appropriate standard OMOP concept IDs across different domains (e.g., SNOMED codes for “Condition” domain, and RxNorm for active ingredients in the “Drug” domain). Thus, the appropriate OMOP concept IDs for bleeding were translated from validated ICD-9-CM and ICD-10-CM codes for bleeding [25, 26], excluding trauma-related bleeding events, using the concept set builder toolkit in the Observational Health Data Sciences and Informatics ATLAS program [27] and applying the recommended practices to define ADEs [28]. The OMOP concept IDs are presented in eTable 2 of the Supplement.

### Features

A total of 88 features were selected according to clinicians’ advice and literature review [29]. We included sociodemographic information, past medical history, substance use behaviors, and concurrent drug use as features in all models. The following three groups of features, totaling 16 features, were specific to the combined SSRI models: current SSRI use, SSRI used just before the newly prescribed SSRI, and the number of prior SSRI switches. Sources of features were longitudinal EHR data as well as cross-sectional survey data collected during *AoU* recruitment. All EHR-derived features, other than concurrent drug use, were determined during the period prior to index date. Concurrent drug use holds the value between 0 and 1, where 0 indicates no overlap in drug use while 1 indicates 100% overlap in drug use between drug features and researched drugs during the follow-up period. The features are listed in Table 1 but more detailed information regarding the source of features (EHR or survey) and, if applicable, the corresponding OMOP concept IDs are included in eTable 3 of the Supplement.

**Table 1:** The list of a priori selected features and their respective feature clusters

Feature clusters	Features	Included in which models
Demographics	Sex at birth (male, female), age at index date	All
Race/ethnicity	Hispanic, Asian, Black or African American, White	All
Comorbidities	Hypertension, renal disease, liver disease, cerebrovascular disease, bleeding disorder, organ transplant	All
Bleeding history	History of bleeding	All
Socioeconomic	Highest education level achieved (no high school degree, high school graduate, college 1–3 years, college 4 or more years or advanced degree), employed for wages or self-employed, annual household income (< 10 k, 10-25 k, 25-35 k, 35-50 k, 50-75 k, 75-100 k, 100-150 k, 150-200 k, ≥ 200 k), health insurance	All
Alcohol use	At least once: alcohol drinking	All
Smoking	100 cigarettes lifetime At least once: cigar smoking, electronic smoking, hookah smoking, smokeless tobacco	All
Recreational drug use	At least once: cocaine, hallucinogens, inhalants, marijuana, methamphetamine, prescription opioids, stimulants, sedatives, street opioids	All
Concurrent drug use: antithrombotics	Clopidogrel, warfarin, apixaban, rivaroxaban, dabigatran, edoxaban, ticagrelor, prasugrel, dipyridamole, ticlopidine, eptifibatide, aspirin (low dose)	All
Concurrent drug use: non-steroidal anti-inflammatory drugs (NSAIDs)	Aspirin (high dose), ibuprofen, indomethacin, naproxen, mefenamic acid, ketorolac, meloxicam, celecoxib, diclofenac	All
Concurrent drug use: glucocorticoids	Prednisone, prednisolone, methylprednisolone, dexamethasone, hydrocortisone	All
Health literacy	Brief Health Literacy Screen	All
Current SSRI use	Citalopram, escitalopram, fluoxetine, fluvoxamine, paroxetine, sertraline, vortioxetine	Combined SSRIs models
SSRI used just before the newly prescribed SSRI	None, citalopram, escitalopram, fluoxetine, fluvoxamine, paroxetine, sertraline, vortioxetine	Combined SSRIs models
Number of prior SSRI switches	Number of switches	Combined SSRIs models

*Abbreviations*: *NSAIDs* non-steroidal anti-inflammatory drugs, *SSRI* selective serotonin reuptake inhibitor

### Machine learning approaches

We developed and validated four different ML algorithms commonly used in binary classification tasks: logistic regression (LR), decision trees (DT), random forest (RF), and extreme gradient boost (XGBoost). The selection of the ML algorithms was informed by previous ML-based studies in ADE prediction [30]. LR was included as it is the dominate model used on EHR data for predicting ADEs and in other clinical prediction models [30]. Each dataset was randomly divided into training and test data using a ten-fold stratified cross validation method. Missing data were imputed using the Scikit-Learn [31] SimpleImputer method with the mode and median being used for categorical and continuous features, respectively. To address the concerns of imbalanced datasets, the effectiveness of randomly oversampling the minority classification was tested for each dataset and ML model. The descriptions of the ML algorithms are provided in eMethods of the Supplement.

### Prediction performance evaluation

To assess the performance of each prediction model, we used the area under the receiver operating characteristic curve statistic (AUC score), as well as performance metrics including sensitivity, specificity, positive predictive value, negative predictive value, positive likelihood ratio, negative likelihood ratio and F1 score. These metrics were assessed at the optimized threshold defined by the Youden’s index [32].

### Feature cluster importance and clinical significance

We calculated feature importance based on a combination of statistical and pharmacological information. Features that are correlated with another feature are subject to having their feature importance diminished and overlooked. To reduce likelihood of this occurrence, we first grouped the features into clusters based on pharmacological and clinical relationships, then interpreted the clinical importance of related features in predicting bleeding events (Table 1). This was accomplished by iteratively removing each cluster individually with replacement to quantify the impact on the AUC score for each ML model. Cluster removals that resulted in a > 0.01 decline in AUC score were classified as important [33]. We defined clinically significant feature clusters based on a stricter threshold of resulting in a > 0.01 decline in AUC score among 3 out of 4 ML models (frequency ≥ 0.75).

### Statistical analysis

We summarized the total number of participants and bleeding events with counts and percentages as descriptive statistics. For model performance metrics, we focused on reporting the AUC and Youden’s index optimized sensitivity and specificity. The importance of each feature cluster was summarized as radar plots based on the frequency (range: 0–1) of resulting in a > 0.01 decline in AUC score across all models for each cohort. Data were accessed with Google BigQuery and analyzed using Python version 3.7.12 in an integrated Jupyter Notebook environment. Results were reported in compliance with the *AoU* Data and Statistics Dissemination Policy prohibiting the display of participant counts ranging from 1 to 20.

## Results

### Descriptive statistics

At the time of analysis, there were 329,038 participants in the registered tier *AoU* dataset version R2021Q3R2, with up to 271,124 participants having both EHR and survey data. We identified 2,159 participants with reliable data for clopidogrel exposure, 1,855 for warfarin, 3,151 for citalopram, 2,597 for escitalopram, 2,719 for fluoxetine, 117 for fluvoxamine, 1,100 for paroxetine, 4,052 for sertraline and 149 for vortioxetine.

The average age at index was 49.4 years for SSRIs, compared to 63.1 for clopidogrel and 60.2 for warfarin. More female participants received SSRIs, except for citalopram which included a much larger proportion of male than female participants (65.1% vs 33.0%). For all cohorts, there was a much larger proportion of White participants, 69.8% (paroxetine) to 81.2% (vortioxetine), compared to other races. The descriptive statistics for each cohort are summarized in Table 2.

**Table 2:** Descriptive statistics of each drug cohorts

	**Clopidogrel**	**Warfarin**	**Citalopram**	**Escitalopram**	**Fluoxetine**	**Fluvoxamine**	**Paroxetine**	**Sertraline**	**Vortioxetine**	**Combined SSRIs**
**Cohort size**	2,159	1,855	3,151	2,597	2,719	117	1,100	4,052	149	10,362
**Sex at birth, n (%)**										
Male	1,248 (57.8)	909 (49.0)	2,052 (65.1)	697 (26.8)	834 (30.7)	42 (35.9)	364 (33.1)	1,332 (32.9)	37 (24.8)	4,390 (31.1)
Female	867 (40.2)	906 (48.8)	1,040 (33.0)	1,862 (71.7)	1,847 (67.9)	74 (63.3)	721 (65.6)	2,668 (65.8)	110 (73.8)	9,508 (67.4)
**Age, mean (SD)**										
At index	63.1 (10.4)	60.2 (11.6)	50.4 (14.3)	50.2 (15.5)	48.1 (14.8)	43.3 (14.2)	51.6 (13.3)	49.0 (15.6)	48.8 (13.4)	49.4 (15.0)
At survey	66.9 (10.2)	66.0 (11.6)	55.9 (15.1)	52.7 (16.0)	52.7 (15.8)	46.4 (15.1)	57.1 (14.1)	52.3 (16.3)	49.6 (13.6)	53.6 (15.8)
**Race, n (%)**										
White	1,571 (72.8)	1,370 (73.9)	2,273 (72.1)	2,003 (77.1)	1,999 (73.5)	92 (78.6)	768 (69.8)	2,928 (72.3)	121 (81.2)	10,341 (73.4)
Black or African American	387 (17.9)	324 (17.5)	590 (18.7)	356 (13.7)	484 (17.8)	≤ 20	222 (20.2)	741 (18.3)	≤ 20	2,468 (17.5)
Asian	≤ 20	≤ 20	≤ 20	27 (1.0)	≤ 20	0 (0.0)	≤ 20	36 (0.9)	≤ 20	104 (0.7)
**Hispanic/Latino, n (%)**	91 (4.2)	76 (4.1)	112 (3.6)	93 (3.6)	99 (3.6)	≤ 20	51 (4.6)	175 (4.3)	≤ 20	538 (3.8)
**Highest education level, n (%)**										
No high school degree	117 (5.4)	84 (4.5)	133 (4.2)	94 (3.6)	122 (4.5)	≤ 20	47 (4.3)	183 (4.5)	≤ 20	593 (4.2)
High school graduate	479 (22.2)	407 (21.9)	713 (22.6)	490 (18.9)	578 (21.3)	≤ 20	262 (23.8)	871 (21.5)	27 (18.1)	3,018 (21.4)
College 1–3 years	735 (34.0)	605 (32.6)	1,057 (33.5)	827 (31.8)	899 (33.1)	38 (32.5)	262 (23.8)	1,367 (33.7)	50 (33.6)	4,725 (33.5)
College 4 years or more or advanced degree	771 (35.7)	710 (38.3)	1,163 (36.9)	1,139 (43.9)	1,061 (39.0)	56 (47.9)	385 (35.0)	1,456 (35.9)	68 (45.6)	5,384 (38.2)
**Health insurance, n (%)**	2,039 (94.4)	1,769 (95.4)	2,976 (94.5)	2,478 (95.4)	2,554 (93.9)	115 (98.3)	1,021 (92.8)	3,835 (94.6)	142 (95.3)	13,313 (94.4)
**Employed for wages or self-employed, n (%)**	492 (22.8)	405 (21.8)	1,103 (56.9)	1,107 (42.6)	1,045 (38.4)	41 (35.0)	372 (33.8)	1,429 (35.3)	52 (34.9)	5,246 (37.2)
**Annual household income, n (%)**										
Less 10 k	190 (8.8)	176 (9.5)	439 (13.9)	344 (13.3)	421 (15.5)	29 (24.8)	158 (14.4)	578 (14.3)	23 (15.4)	2,051 (14.6)
10-25 k	404 (18.7)	330 (17.8)	561 (17.8)	424 (16.3)	504 (18.5)	26 (22.2)	237 (21.6)	700 (17.3)	34 (22.8)	2,525 (17.9)
25-35 k	240 (11.1)	187 (10.1)	334 (10.6)	280 (10.8)	268 (9.9)	≤ 20	98 (8.9)	430 (10.6)	≤ 20	1,436 (10.2)
35-50 k	247 (11.4)	226 (12.2)	337 (10.7)	258 (9.9)	291 (10.7)	≤ 20	116 (10.6)	417 (10.3)	≤ 20	1,454 (10.3)
50-75 k	277 (12.8)	231 (12.5)	383 (12.2)	338 (13.0)	321 (11.8)	≤ 20	140 (12.7)	529 (13.1)	≤ 20	1,760 (12.5)
75-100 k	172 (8.0)	157 (8.5)	279 (8.9)	235 (9.1)	233 (8.6)	≤ 20	88 (8.0)	355 (8.8)	≤ 20	1,238 (8.8)
100-150 k	158 (7.3)	157 (8.5)	257 (8.2)	236 (9.1)	195 (7.2)	0 (0.0)	69 (6.3)	296 (7.3)	≤ 20	1,096 (7.8)
150-200 k	40 (1.9)	37 (2.0)	84 (2.7)	84 (3.2)	79 (2.9)	≤ 20	25 (2.3)	111 (2.7)	≤ 20	393 (2.8)
More 200 k	54 (2.5)	41 (2.2)	77 (2.4)	96 (3.7)	75 (2.8)	≤ 20	27 (2.5)	105 (2.6)	≤ 20	395 (2.8)

*Abbreviations*: *SD* standard deviation, *SSRIs* selective serotonin reuptake inhibitors

Results were reported in compliance with the AoU Data and Statistics Dissemination Policy prohibiting the display of participant counts ranging 1 to 20

The proportion of bleeding events after drug exposure was 10.8% for clopidogrel and 15.8% for warfarin. Across individual SSRIs, the percentages of bleeding events ranged from 6.0% in escitalopram to 9.1% in citalopram. When combining all the SSRIs into a single combined SSRI cohort, there were 10,362 participants exposed to at least one of the seven SSRIs, with 9.6% experiencing a bleeding event upon SSRI exposure. These statistics are summarized in Table 3.

**Table 3:** Cohort size, number of bleeding events, and best model performance metrics for each drug cohorts

	**Total number of patients, N**	**Number of bleeding events, n (%)**	**Best models based on largest AUC score**			
			**ML model**	**AUC**	**YI optimized sensitivity**	**YI optimized specificity**
Clopidogrel	2,159	234 (10.8)	LR	0.638	64.4%	59.5%
Warfarin	1,855	293 (15.8)	XGBoost	0.682	69.0%	61.0%
Citalopram	3,151	286 (9.1)	RF	0.698	67.8%	66.7%
Escitalopram	2,597	156 (6.0)	RF	0.656	67.3%	59.1%
Fluoxetine	2,719	226 (8.3)	DT	0.664	36.8%	85.4%
Fluvoxamine^a^	117	≤ 20	-	-	-	-
Paroxetine	1,100	97 (8.8)	RF	0.632	58.9%	63.2%
Sertraline	4,052	336 (8.3)	RF	0.665	66.8%	61.9%
Vortioxetine^a^	149	≤ 20	-	-	-	-
Combined SSRI	10,362	996 (9.6)	XGBoost	0.688	57.9%	70.6%

*Abbreviations*: *AUC* area under the receiver operating characteristic curve statistic, *DT* decision tree, *LR* logistic regression, *ML* machine learning, *RF* random forest, *XGBoost* extreme gradient boosting, *YI* Youden’s index

Results were reported in compliance with the AoU Data and Statistics Dissemination Policy prohibiting the display of participant counts ranging 1 to 20

^a^The models for fluvoxamine and vortioxetine were excluded due to the small number of participants in the cohorts relative to other drugs

### Model performance

**Fig. 1:**
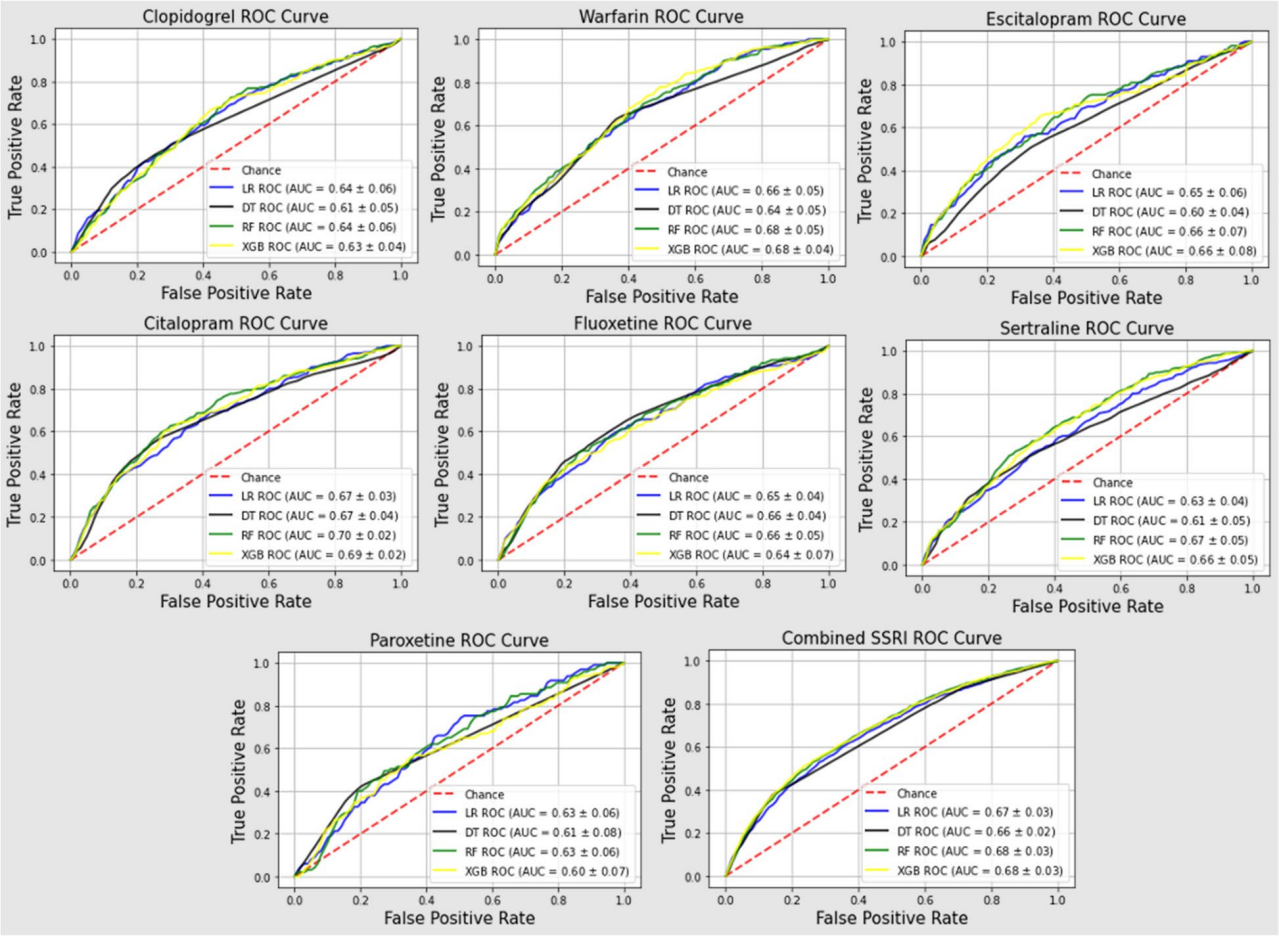
Receiver operator curves with area under the curve (AUC) scores. Higher AUC score represents better model performance. Baseline characteristics of participants in each cohort served as features for bleeding event prediction with logistic regression (LR), decision tree (DT), random forest (RF) and extreme gradient boosting (XGB) machine learning models

Datasets without feature selection and oversampling of the minority class were selected as primary inputs for each of the ML models. A total of 40 models, four for each of the 10 cohorts, were developed. The models for fluvoxamine and vortioxetine were excluded due to the small number (*n* < 150) of participants in the cohorts relative to other drugs. Nevertheless, these participants were still included in the combined SSRI cohort. Table 3 summarizes the best performing model with AUC score and the corresponding Youden’s index-optimized sensitivity and specificity for each drug cohort. The hyperparameters of the best performing models are summarized in eTable 4 of the Supplement. Figure 1 summarizes the AUC score for each individual drug as well as the dataset with all SSRIs combined. The AUC scores and other metrics for each ML model and drug for datasets with feature selection and an oversampling of the minority class can be found in eTables 5–13 in the Supplement.

### Feature clustering and importance

**Fig. 2:**
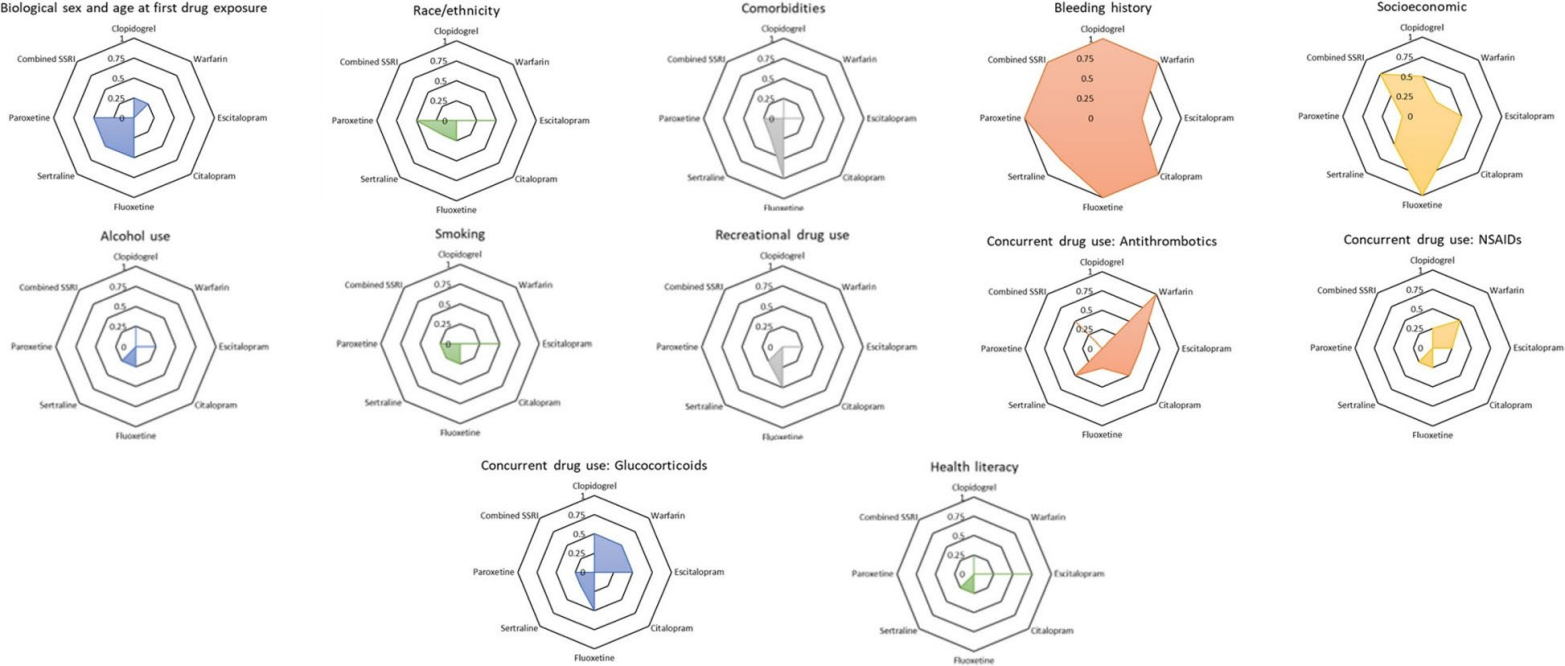
The importance of each feature cluster was summarized as radar plots based on the frequency (range: 0–1) of resulting in a > 0.01 decline in AUC score across four machine learning (ML) models (logistic regression, decision tree, random forest, and extreme gradient boosting) for each cohort. The larger the chart area, the more important the feature cluster was across all cohorts (0.25 = important in one ML model, 0.50 = important in two ML models, 0.75 = important in three ML models, 1 = important for all four ML models)

In total, there were 15 clusters summarizing 88 features (Table 1). For this analysis, three clusters comprising 16 features (current SSRI use, SSRI used just before the newly prescribed SSRI, and the number of prior SSRI switches) were not examined as they were only present in the combined SSRI models. Bleeding history and socioeconomic status were the top two most important clusters across all cohorts (Fig. 2). In fact, bleeding history feature removal was found to cause > 0.01 decline in AUC scores across all four ML models (LR, DT, RF and XGBoost) for all cohorts except for sertraline (3 models, frequency: 0.75), and escitalopram (2 models, frequency: 0.5) (Fig. 2).

### Clinically significant feature clusters

Bleeding history was a clinically significant feature for all drugs except for escitalopram. For escitalopram, health literacy is the only clinically significant feature. Antithrombotics were clinically significant for warfarin, while features for socioeconomic status (highest education level, employment status, annual household income, and health insurance) were significant for fluoxetine and combined SSRIs cohorts (Table 4).

**Table 4:** Clinically significant feature clusters for each drug cohort

Cohort	Clinically significant feature clusters^a^
Clopidogrel	Bleeding history
Warfarin	Bleeding history, antithrombotics
Escitalopram	Health literacy
Citalopram	Bleeding history
Fluoxetine	Bleeding history, socioeconomic
Sertraline	Bleeding history
Paroxetine	Bleeding history
Combined SSRIs	Bleeding history, socioeconomic

^a^ Clinically significant feature clusters resulted in a > 0.01 decline in AUC score among 3 out of 4 machine learning models (frequency ≥ 0.75)

## Discussion

We developed ML models with close to moderate predictive performance for SSRI-associated bleeding using data from the NIH *AoU* Research Program as part of what will be a larger precision medicine endeavor. The *AoU* database allows us to create models incorporating not only clinical information from the EHR but also sociodemographic characteristics through survey data including income, health literacy, and education level. More importantly, we created our models with the goal of eventually implementing them in clinical practice to allow for evaluation of patient-specific factors and individualized bleeding risk scores for each SSRI to select therapy with the lowest possible risk. Thus, most of our features were selected to ensure that they can be feasibly obtained in clinical settings.

Multiple meta-analyses have demonstrated an augmented risk of gastrointestinal (GI) bleeding with SSRIs, especially when taken concurrently with a non-steroidal anti-inflammatory drug (NSAID) [34–36]. Another meta-analysis demonstrated an increased risk of intracerebral and intracranial hemorrhage (ICH) with SSRIs, albeit these bleeding events were rare [37]. There was an estimated a 36% increase in non-specific, global bleeding risk from SSRI treatment [10]. Despite the literature establishing SSRI bleeding risk, studies have not extensively examined actionable risk factors to prevent bleeding ADEs. To our knowledge, this is the first ML prediction model developed specifically for bleeding events associated with SSRIs.

Prior bleeding history was identified as clinically significant in almost all drug cohorts, except escitalopram, although bleeding history remains arguably important as significant changes in AUC were found in two out of its four ML escitalopram models. This is unsurprising as bleeding history is a component of bleeding risk stratification tools for other clinical settings such as HAS-BLED, RIETE, and VTE-BLEED [29, 38]. Further, this evidenced the importance of evaluating predisposing risk factors to bleeding prior to SSRI prescribing. Socioeconomic status was identified as a clinically important feature cluster in the fluoxetine cohort and the combined SSRI cohort. This is an important finding as hospital admissions due to antidepressant-related ADE were also identified to be higher in patients from low-income areas [39] and the need for use of antidepressants may be higher in low-income populations [40]. Patients with low socioeconomic status received low-quality health care coupled with unstandardized care coordination which has caused suboptimal use of medications [41, 42]. Health literacy based on survey data was also deemed clinically significant in the escitalopram cohort. Health literacy affects a person’s capability to interpret and execute health information [43, 44]. Patients with poorer health literacy frequently misunderstood drug information, including over-the-counter drugs [45, 46], which could lead to unintended yet preventable adverse drug events especially in underserved communities [47, 48]. These support the need to examine sociodemographic factors for evaluation of ADE risk at the time of prescribing, as well as interventions to improve patient understanding of their medications.

Surprisingly, use of concurrent antithrombotics was defined as clinically important only for the warfarin cohort and concurrent NSAID use was not noted to be clinically significant in our ML models which is inconsistent with previous studies evaluating bleeding risk with SSRIs [34–36, 49]. This may be explained by the incomplete nature of EHR data (which was used to quantify these features) as a consequence of patients' visits to multiple health institutions for care and prescription filling. This presents a significant challenge in the implementation of clinical prediction models in routine clinical practice, especially if the use of real-world EHR data for feature extraction and engineering is desired. Nevertheless, there is great research potential in this field if clinicians and health informaticians work together. For example, clinicians routinely perform medication reconciliation, a process involving the comparison of a patient's medical record to an external list of medications obtained from various sources to determine the most precise and complete list of all medications, including their names, dosages, frequencies, and routes of administration. Health informaticians design and maintain the electronic health system and have expertise in extracting real-world EHR data to train and implement clinical prediction models. Collaborations between both professionals can facilitate the development of clinically actionable prediction models and optimize patient health outcomes. Therefore, we emphasize that our findings do not conclude that concurrent medications and comorbidities are less significant for predicting ADEs. Rather, it uncovers the limitations with EHR data, barriers with training and implementing clinical prediction models in real-world practice, and other modifiable risk factors that clinicians should consider addressing.

While the AUC scores and Youden’s index-optimized sensitivity and specificity for each drug cohort are modest, the performances of models established from this study are comparable to those of previously validated prediction models for clinically relevant bleeding. In the AMADEUS study, CHADS_2_, CHA_2_DS_2_-VASc and HAS-BLED scores were used to determine predictive value for bleeding for enrolled patients [50]. The best performing model, and only one of the three recommended to perform bleeding risk assessment, was HAS-BLED, which demonstrated a modest performance in predicting clinically relevant bleeding, with an AUC of 0.60. Of note, prediction of bleeding events in this study was in patients with atrial fibrillation being treated with anticoagulants; thus, its findings are likely not directly comparable to ours. Nevertheless, this illustrates that our models demonstrate at least comparable performance to currently utilized prediction models in clinical settings.

Developing ML models on EHR data to predict ADEs has been of interest to the research community. Zhao et al*.* tested multiple ML models including regression, decision trees, AdaBoost, and Random Forest on EHR data to predict ADEs [51]. They showed that, with careful feature selection, ML models can achieve promising accuracy as high as 85% in predicting ADEs [51]. Given the widespread understanding of regression models across health disciplines, these models are predominantly used on EHR data for predicting ADEs [30], with LR found to perform similar to other ML models across multiple clinical prediction studies [52] as further verified by our findings. Future ADE studies can continue exploring with LR using more optimal EHR features, such as the most recent laboratory results and current medication lists at time of office or pharmacy visit.

This study does have some limitations. As explained previously, there are inherent limitations when using EHR databases retrospectively for ADE research. Selection of participants and identification of ADEs is challenging, as it is difficult to ascertain information necessary for thorough causality assessment. Poor quality data collected from EHR sources designed for non-research methods, or missing data, may lead to selection bias and information bias. Therefore, we applied recommended practices to address these inherent limitations, employing strategies such as defining the index date as the first drug exposure date to reduce the risk of immortal time bias [28]. We also designed the follow-up period carefully and treated drug exposure as a time-varying feature, considering factors such gaps in medication records and initiation of other drugs, rather than assuming initial exposure remains the same throughout the follow-up period. Feature selection and clusters were determined a priori, which could have excluded important features identifiable with empirical methods, while the definition of clinically significant features requires optimization. Nonetheless, the rich data made available by the *AoU* program allow us to make robust predictions with reasonable sample sizes while performing hypothesis-generating research for further evaluation with prospective studies.

## Conclusion

We observed that bleeding history, socioeconomic status, and health literacy were important factors that may predict bleeding associated with SSRI use. This work contributes to the larger conversation on judicious use of medications and the importance of optimizing non-drug treatment modalities such as psychotherapy, lifestyle management, and psychosocial interventions whenever possible. Public health interventions that focus on increasing health literacy and provide more health care resources in low-income neighborhoods will go a long way to reduce adverse events worldwide. Although our models performed better than many existing clinical models, we expect improvements in the performance of our current models with the inclusion of genomic features and pharmacokinetic drug interactions [53], alongside optimization of real-world medication and health outcomes using EHR. We will also explore with deep learning models, such as recurrent neural networks, to better capture the granularity of medication changes (dose and frequency) that may be important for ADE prediction.

## Supplementary Information

**Additional file 1: eTable 1.** Drug concept IDs.** eTable 2.** Bleeding algorithm.** eTable 3.** Features.** eTable 4.** Hyperparameters of best models.** eTable 5.** AUC score for all models.** eTable 6.** Clopidogrel performance statistics.** eTable 7.** Warfarin performance statistics.** eTable 8.** Escitalopram performance statistics.** eTable 9.** Citalopram performance statistics.** eTable 10.** Fluoxetine performance statistics.** eTable 11.** Sertraline performance statistics.** eTable 12.** Paroxetine performance statistics.** eTable 13.** Combined SSRI performance statistics.

## Data Availability

The *All of Us* Research Program data used in this study are considered an open-source database.

## References

[1] Santo L, Okeyode T. National Ambulatory Medical Care Survey: 2018 National Summary Tables. Published 2018. https://www.cdc.gov/nchs/data/ahcd/namcs_summary/2018-namcs-web-tables-508.pdf. Accessed 14 July 2022.

[2] Shehab N, Lovegrove MC, Geller AI, Rose KO, Weidle NJ, Budnitz DS (2016). US emergency department visits for outpatient adverse drug events, 2013–2014. JAMA - J Am Med Assoc.

[3] Sultana J, Cutroneo P, Trifirò G (2013). Clinical and economic burden of adverse drug reactions. J Pharmacol Pharmacother.

[4] Weiss AJ, Freeman WJ, Heslin KC, Barrett ML. Statistical Brief #234: Adverse Drug Events in U.S. Hospitals, 2010 Versus 2014. Agency for Healthcare Research and Quality. Published 2018. https://www.hcup-us.ahrq.gov/reports/statbriefs/sb234-Adverse-Drug-Events.jsp. Accessed July 14, 2022.

[5] Aspden P, Wolcott J, Bootman JL, Cronenwett L, eds; Institute of Medicine, Committee on Identifying and Preventing Medication Errors. Washington DC: National Academies Press; 2007. ISBN 0309101476.

[6] Falconer N, Barras M, Cottrell N (2018). Systematic review of predictive risk models for adverse drug events in hospitalized patients. Br J Clin Pharmacol.

[7] Cheng CM (2011). Hospital systems for the detection and prevention of adverse drug events. Clin Pharmacol Ther.

[8] Mack MR, Kim BS (2020). A precision medicine–based strategy for a severe adverse drug reaction. Nat Med.

[9] Alessandrini M, Chaudhry M, Dodgen TM, Pepper MS (2016). Pharmacogenomics and global precision medicine in the context of adverse drug reactions: Top 10 opportunities and challenges for the next decade. Omi A J Integr Biol.

[10] Laporte S, Chapelle C, Caillet P (2017). Bleeding risk under selective serotonin reuptake inhibitor (SSRI) antidepressants: A meta-analysis of observational studies. Pharmacol Res.

[11] Bixby AL, VandenBerg A, Bostwick JR (2019). Clinical Management of Bleeding Risk With Antidepressants. Ann Pharmacother.

[12] Chu A, Wadhwa R. Selective Serotonin Reuptake Inhibitors. StatPearls Publishing; 2022. https://www.ncbi.nlm.nih.gov/books/NBK554406/32119293

[13] Kalbouneh HM, Toubasi AA, Albustanji FH, Obaid YY, Al-Harasis LM (2022). Safety and efficacy of SSRIs in improving poststroke recovery: a systematic review and meta-analysis. J Am Heart Assoc.

[14] Hirsch M, Birnbaum RJ. Selective serotinin reuptake inhibitors: pharmacology, administration, and side effects. In: UptoDate, Roy-Byrne P, editor. UptoDate. Waltham.

[15] Wägner A, Montero D, Mårtensson B, Siwers B, Åsberg M (1990). Effects of fluoxetine treatment of platelet 3H-imipramine binding, 5-HT uptake and 5-HT content in major depressive disorder. J Affect Disord.

[16] Hergovich N, Aigner M, Eichler HG, Entlicher J, Drucker C, Jilma B (2000). Paroxetine decreases platelet serotonin storage and platelet function in human beings. Clin Pharmacol Ther.

[17] Javors MA, Houston JP, Tekell JL, Brannan SK, Frazer A (2000). Reduction of platelet serotonin content in depressed patients treated with either paroxetine or desipramine. Int J Neuropsychopharmacol.

[18] De Abajo FJ (2011). Effects of selective serotonin reuptake inhibitors on platelet function: Mechanisms, clinical outcomes and implications for use in elderly patients. Drugs Aging.

[19] Andrade C, Sandarsh S, Chethan KB, Nagesh KS (2010). Serotonin reuptake inhibitor antidepressants and abnormal bleeding: A review for clinicians and a reconsideration of mechanisms. J Clin Psychiatry.

[20] Zanger UM, Schwab M (2013). Cytochrome P450 enzymes in drug metabolism: regulation of gene expression, enzyme activities, and impact of genetic variation. Pharmacol Ther.

[21] Syrowatka A, Song W, Amato MG (2022). Key use cases for artificial intelligence to reduce the frequency of adverse drug events: a scoping review. Lancet Digit Heal.

[22] Collins FS, Varmus H (2015). A New Initiative on Precision Medicine. N Engl J Med.

[23] Denny JC, Rutter JL, Goldstein DB, Philippakis A, Smoller JW, Jenkins G, Dishman E. The “All of Us” Research Program. N Engl J Med. 2019;381(7):668-76. 10.1056/NEJMsr1809937. 10.1056/NEJMsr1809937 PMC8291101 31412182

[24] Hripcsak G, Duke JD, Shah NH (2015). Observational Health Data Sciences and Informatics (OHDSI): opportunities for observational researchers. Stud Health Technol Inform.

[25] Cunningham A, Stein CM, Chung CP, Daugherty JR, Smalley WE, Ray WA (2011). An automated database case definition for serious bleeding related to oral anticoagulant use. Pharmacoepidemiol Drug Saf.

[26] Siontis KC, Zhang X, Eckard A (2018). Outcomes associated with apixaban use in patients with end-stage kidney disease and atrial fibrillation in the United States. Circulation.

[27] Observational Health Data Sciences and Informatics (OHDSI). ATLAS. https://atlas.ohdsi.org/

[28] Ng DQ, Dang E, Chen L (2021). Current and recommended practices for evaluating adverse drug events using electronic health records: a systematic review. Jaccp J Am Coll Clin Pharm.

[29] Pisters R, Lane DA, Nieuwlaat R (2010). A novel user-friendly score (HAS-BLED) to assess 1-year risk of major bleeding in patients with atrial fibrillation: The Euro heart survey. Chest.

[30] Kim HR, Sung M, Park JA (2022). Analyzing adverse drug reaction using statistical and machine learning methods: A systematic review. Medicine (Baltimore).

[31] Pedregosa F, Varoquaux G, Gramfort A (2011). Scikit-learn: machine learning in python. J Mach Learn Res.

[32] Fluss R, Faraggi D, Reiser B (2005). Estimation of the Youden Index and its associated cutoff point. Biometrical J.

[33] Lyu J, Li JJ, Su J (2020). DORGE: Discovery of Oncogenes and tumoR suppressor genes using Genetic and Epigenetic features. Sci Adv.

[34] Jiang H-Y, Chen H-Z, Hu X-J (2015). Use of selective serotonin reuptake inhibitors and risk of upper gastrointestinal bleeding: a systematic review and meta-analysis. Clin Gastroenterol Hepatol.

[35] Anglin R, Yuan Y, Moayyedi P, Tse F, Armstrong D, Leontiadis GI (2014). Risk of upper gastrointestinal bleeding with selective serotonin reuptake inhibitors with or without concurrent nonsteroidal anti-inflammatory use: A systematic review and meta-analysis. Am J Gastroenterol.

[36] Loke YK, Trivedi AN, Singh S (2008). Meta-analysis: Gastrointestinal bleeding due to interaction between selective serotonin uptake inhibitors and non-steroidal anti-inflammatory drugs. Aliment Pharmacol Ther.

[37] Hackam DG, Mrkobrada M (2012). Selective serotonin reuptake inhibitors and brain hemorrhage: a meta-analysis. Neurology.

[38] Lecumberri R, Jiménez L, Ruiz-Artacho P (2021). Prediction of major bleeding in anticoagulated patients for Venous Thromboembolism: Comparison of the RIETE and the VTE-BLEED Scores. TH Open.

[39] Parihar HS, Yin H, Gooch JL, Allen S, John S, Xuan J (2017). Trends in hospital admissions due to antidepressant-related adverse drug events from 2001 to 2011 in the U.S. BMC Health Serv Res.

[40] Patel V, Burns JK, Dhingra M, Tarver L, Kohrt BA, Lund C (2018). Income inequality and depression: a systematic review and meta-analysis of the association and a scoping review of mechanisms. World Psychiatry.

[41] Hwang J, Lyu B, Ballew S (2022). The association between socioeconomic status and use of potentially inappropriate medications in older adults. J Am Geriatr Soc Published online.

[42] Green AJ, Fox KM, Grandy S (2012). Self-reported hypoglycemia and impact on quality of life and depression among adults with type 2 diabetes mellitus. Diabetes Res Clin Pract.

[43] Sarkar U, Karter AJ, Liu JY, Moffet HH, Adler NE, Schillinger D (2010). Hypoglycemia is more common among type 2 diabetes patients with limited health literacy: the diabetes study of Northern California (DISTANCE). J Gen Intern Med.

[44] Hickey KT, Masterson Creber RM, Reading M (2018). Low health literacy: Implications for managing cardiac patients in practice. Nurse Pract.

[45] Wali H, Grindrod K (2016). Don’t assume the patient understands: Qualitative analysis of the challenges low health literate patients face in the pharmacy. Res Soc Adm Pharm.

[46] Kim M, Suh D, Barone JA, Jung SY, Wu W, Suh DC (2022). Health literacy level and comprehension of prescription and nonprescription drug information. Int J Environ Res Public Health.

[47] Rungvivatjarus T, Huang MZ, Winckler B, Chen S, Fisher ES, Rhee KE (2023). Parental factors affecting pediatric medication management in underserved communities. Acad Pediatr.

[48] Gupta V, Shivaprakash G, Bhattacherjee D (2020). Association of health literacy and cognition levels with severity of adverse drug reactions in cancer patients: a South Asian experience. Int J Clin Pharm.

[49] Dalton SO, Johansen C, Mellemkjær L, Nørgård B, Sørensen HT, Olsen JH (2003). Use of selective serotonin reuptake inhibitors and risk of upper gastrointestinal tract bleeding a population-based cohort study. Arch Intern Med.

[50] Apostolakis S, Lane DA, Buller H, Lip GYH (2013). Comparison of the CHADS2, CHA2DS2 -VASc and HAS-BLED scores for the prediction of clinically relevant bleeding in anticoagulated patients with atrial fibrillation: The AMADEUS trial. Thromb Haemost.

[51] Zhao J, Henriksson A, Asker L, Boström H (2015). Predictive modeling of structured electronic health records for adverse drug event detection. BMC Med Inform Decis Mak.

[52] Christodoulou E, Ma J, Collins GS, Steyerberg EW, Verbakel JY, Van Calster B (2019). A systematic review shows no performance benefit of machine learning over logistic regression for clinical prediction models. J Clin Epidemiol.

[53] Ramirez AH, Gebo KA, Harris PA (2021). Progress with the all of us research program: opening access for researchers. JAMA - J Am Med Assoc.

