# Supplementary material for: Using machine learning to develop a clinical prediction model for SSRI-associated bleeding: a feasibility study
Source: BMC Med Inform Decis Mak. 2023 Jun 11;23:105. doi: 10.1186/s12911-023-02206-3 (PMC10257821; doi:10.1186/s12911-023-02206-3)
Supplement: Supplementary file 1 — Additional file 1: eTable 1. Drug concept IDs. eTable 2. Bleeding algorithm. eTable 3. Features. eTable 4. Hyperparameters of best models. eTable 5. AUC score for all models. eTable 6. Clopidogrel performance statistics. eTable 7. Warfarin performance statistics. eTable 8. Escitalopram performance statistics. eTable 9. Citalopram performance statistics. eTable 10. Fluoxetine performance statistics. eTable 11. Sertraline performance statistics. eTable 12. Paroxetine performance statistics. eTable 13. Combined SSRI performance statistics. [file 12911_2023_2206_MOESM1_ESM.docx]

***Supplement of “Using Machine Learning to Develop a Clinical Prediction Model for SSRI-associated bleeding: a feasibility study”, Goyal et al., 2022.***

**eMethods**

1. **Logistic regression**

Logistic Regression (LR) is an especially useful method when predicting a binary outcome and is a method that is commonly used in medical applications.^1,2^ It is considered to be one of the simplest supervised ML algorithms that can be used for classification problems. We also used LR with an elastic net penalty^3^ as a method in which to perform feature selection on the dataset, where features with coefficients greater than zero were considered important.^4^ The benefit of LR is the ease in which the model can be trained and interpreted. The accuracy of each model was adjusted with various hyperparameter tuning using Scikit-Learn’s LR library.^5^

1. **Decision Trees**

Decision trees (DT) are a non-parametric supervised ML model that are ideal for classification tasks using simple decision rules.^6,7^ DTs are ideal in handling data with mixed data types (continuous and categorical) while increasing interpretability for splits at each node and validation through multiple statistical tests. Minimizing error can be controlled by tuning numerous parameters that adjust the depth and complexity of the decision tree. Each DT model’s hyperparameters were iteratively adjusted with the combination of parameters that yielded the greatest accuracy and lowest error being empirically chosen. Each model was also protected against overfitting by using a validation dataset. We used the DT Classifier library from the Scikit-Learn open-source ML Python package.^5^

1. **Random Forest**

Random Forest (RF) is an ensemble-based ML method that utilizes a multitude of DTs to perform the classification task.^8–10^ It uses the bootstrapping statistical resampling technique, which involves random sampling of the data with replacement. Further, RFs use the bagging technique, also known as bootstrap aggregating, which combines the predictions of each individual model created by the bootstrapping. This method also incorporates feature randomness to reduce the correlation among individual DTs, ensuring interactions between features with low predictive importance are observed, which could increase the accuracy of the prediction. While RFs are more complex than DTs, this method minimizes the variance and the risk of overfitting. Implementation and accuracy of the RF model was iteratively controlled through multiple tuning hyperparameters that adjusted the number of trees, the depth of each tree, and the overall complexity of each tree. We implemented the RF Classifier library from the Scikit-Learn open-source ML Python package.^5^

1. **Extreme Gradient Boosting**

Extreme Gradient Boosting (XGBoost) is an open-source ML software library that utilizes parallel tree boosting for numerous prediction tasks, such as classification, to improve computational processing speed and accuracy.^11,12^ XGBoost is an ensemble consisting of numerous decision trees that follow the boosting method. Boosting involves combining a single weak DT with other weak DTs to ultimately create a more accurate model. Gradient boosting incorporates the gradient descent algorithm where the error is minimized by using the previous model’s residuals to fit the next model. Gradient boosting also sets a target outcome so that the result from the ensemble is a weighted sum of each model’s predictions. The depth of each decision tree, specification of minimum loss reduction, balance between L1 and L2 regularization, and other factors that affect the accuracy of the model can be adjusted with hyperparameter tuning in the XGBClassifier library from the DMLC XGboost package.^11^

**eReferences**

1. Boo Y, Choi Y. Comparing logistic regression models with alternative machine learning methods to predict the risk of drug intoxication mortality. *Int J Environ Res Public Health*. 2020;17(3). doi:10.3390/ijerph17030897

2. Sperandei S. Understanding logistic regression analysis. *Biochem Medica*. 2014;24(1):12-18. doi:10.11613/BM.2014.003

3. Lyu J, Li JJ, Su J, et al. DORGE: Discovery of Oncogenes and tumoR suppressor genes using Genetic and Epigenetic features. *Sci Adv*. 2020;6(46):6784-6795. doi:10.1126/sciadv.aba6784

4. Guyon I, Gunn S, Nikravesh M, Zadeh LA. *Feature Extraction: Foundations and Applications*. Vol 207. Springer; 2008.

5. Pedregosa F, Varoquaux G, Gramfort A, et al. Scikit-learn: Machine learning in Python. *J Mach Learn Res*. 2011;12.

6. Chern CC, Chen YJ, Hsiao B. Decision tree-based classifier in providing telehealth service. *BMC Med Inform Decis Mak*. 2019;19(1). doi:10.1186/s12911-019-0825-9

7. Webb GI, Fürnkranz J, Fürnkranz J, et al. Decision Tree. In: *Encyclopedia of Machine Learning*. Springer, Boston, MA; 2011:263-267. doi:10.1007/978-0-387-30164-8_204

8. Yang L, Wu H, Jin X, et al. Study of cardiovascular disease prediction model based on random forest in eastern China. *Sci Rep*. 2020;10(1). doi:10.1038/s41598-020-62133-5

9. Asadi S, Roshan SE, Kattan MW. Random forest swarm optimization-based for heart diseases diagnosis. *J Biomed Inform*. 2021;115:103690. doi:10.1016/j.jbi.2021.103690

10. Buhmann MD, Melville P, Sindhwani V, et al. Random Forests. *Encycl Mach Learn*. Published online 2011:828-828. doi:10.1007/978-0-387-30164-8_695

11. Chen T, Guestrin C. XGBoost: A scalable tree boosting system. In: *Proceedings of the ACM SIGKDD International Conference on Knowledge Discovery and Data Mining*. Vol 13-17-Augu. ACM; 2016:785-794. doi:10.1145/2939672.2939785

12. Hou N, Li M, He L, et al. Predicting 30-days mortality for MIMIC-III patients with sepsis-3: a machine learning approach using XGboost. *J Transl Med*. 2020;18(1):462. doi:10.1186/s12967-020-02620-5

**eTable 1: Drug concept IDs**

| **OMOP concept ID** | **Concept code** | **Concept name** | **Vocabulary** | **Domain** | **Class** | **Standard concept caption** |
| --- | --- | --- | --- | --- | --- | --- |
| 1322184 | 32968 | clopidogrel | RxNorm | Drug | Ingredient | Standard |
| 1310149 | 11289 | warfarin | RxNorm | Drug | Ingredient | Standard |
| 797617 | 2556 | citalopram | RxNorm | Drug | Ingredient | Standard |
| 715939 | 321988 | escitalopram | RxNorm | Drug | Ingredient | Standard |
| 755695 | 4493 | fluoxetine | RxNorm | Drug | Ingredient | Standard |
| 751412 | 42355 | fluvoxamine | RxNorm | Drug | Ingredient | Standard |
| 722031 | 32937 | paroxetine | RxNorm | Drug | Ingredient | Standard |
| 739138 | 36437 | sertraline | RxNorm | Drug | Ingredient | Standard |
| 44507700 | 1455099 | vortioxetine | RxNorm | Drug | Ingredient | Standard |
| 32825 | OMOP4976898 | EHR dispensing record | Type Concept | Drug | Drug Type | Standard |
| 32869 | OMOP4976942 | Pharmacy claim | Type Concept | Drug | Drug Type | Standard |
| 38000175 | OMOP4822239 | Prescription dispensed in pharmacy | Type Concept | Drug | Drug Type | Non-standard |
| 581452 | OMOP4822253 | Dispensed in Outpatient office | Type Concept | Drug | Drug Type | Non-standard |
| 32818 | OMOP4976891 | EHR administration record | Type Concept | Drug | Drug Type | Standard |
| 38000180 | OMOP4822244 | Inpatient administration | Type Concept | Drug | Drug Type | Non-standard |
| 38000179 | OMOP4822243 | Physician administered drug (identified as procedure) | Type Concept | Drug | Drug Type | Non-standard |
| 581373 | OMOP4822252 | Physician administered drug (identified from EHR order) | Type Concept | Drug | Drug Type | Non-standard |

**eTable 2: Bleeding algorithm**

| **OMOP concept ID** | **Concept code** | **Concept name** | **Vocabulary** | **Domain** | **Standard concept caption** | **Include** | **Exclude** | **Descendants** |
| --- | --- | --- | --- | --- | --- | --- | --- | --- |
| 4027729 | 12847006 | Acute duodenal ulcer with hemorrhage | SNOMED | Condition | Standard | Yes | No | No |
| 441062 | 87756006 | Acute duodenal ulcer with hemorrhage AND obstruction | SNOMED | Condition | Standard | Yes | No | No |
| 4336230 | 86895006 | Acute duodenal ulcer with hemorrhage AND perforation | SNOMED | Condition | Standard | Yes | No | No |
| 435855 | 51847008 | Acute duodenal ulcer with hemorrhage AND with perforation but without obstruction | SNOMED | Condition | Standard | Yes | No | No |
| 434402 | 66767006 | Acute duodenal ulcer with hemorrhage but without obstruction | SNOMED | Condition | Standard | Yes | No | No |
| 437021 | 41986000 | Acute duodenal ulcer with hemorrhage, with perforation AND with obstruction | SNOMED | Condition | Standard | Yes | No | No |
| 4231580 | 89748001 | Acute gastric ulcer with hemorrhage | SNOMED | Condition | Standard | Yes | No | No |
| 198467 | 46708007 | Acute gastric ulcer with hemorrhage and obstruction | SNOMED | Condition | Standard | Yes | No | No |
| 4169592 | 48974009 | Acute gastric ulcer with hemorrhage and perforation | SNOMED | Condition | Standard | Yes | No | No |
| 199855 | 17067009 | Acute gastric ulcer with hemorrhage AND with perforation but without obstruction | SNOMED | Condition | Standard | Yes | No | No |
| 193795 | 70418001 | Acute gastric ulcer with hemorrhage but without obstruction | SNOMED | Condition | Standard | Yes | No | No |
| 195845 | 195845 | Acute gastric ulcer with hemorrhage, with perforation and with obstruction | SNOMED | Condition | Standard | Yes | No | No |
| 4274491 | 63954007 | Acute gastrojejunal ulcer with hemorrhage | SNOMED | Condition | Standard | Yes | No | No |
| 441063 | 72408002 | Acute gastrojejunal ulcer with hemorrhage and obstruction | SNOMED | Condition | Standard | Yes | No | No |
| 4217947 | 81387001 | Acute gastrojejunal ulcer with hemorrhage and perforation | SNOMED | Condition | Standard | Yes | No | No |
| 441328 | 66673003 | Acute gastrojejunal ulcer with hemorrhage and with perforation but without obstruction | SNOMED | Condition | Standard | Yes | No | No |
| 438468 | 59515005 | Acute gastrojejunal ulcer with hemorrhage but without obstruction | SNOMED | Condition | Standard | Yes | No | No |
| 442314 | 58711008 | Acute gastrojejunal ulcer with hemorrhage, with perforation and with obstruction | SNOMED | Condition | Standard | Yes | No | No |
| 193249 | 2367005 | Acute hemorrhagic gastritis | SNOMED | Condition | Standard | Yes | No | No |
| 4046500 | 12274003 | Acute peptic ulcer with hemorrhage | SNOMED | Condition | Standard | Yes | No | No |
| 23237 | 43406003 | Acute peptic ulcer with hemorrhage AND obstruction | SNOMED | Condition | Standard | Yes | No | No |
| 4006994 | 111353003 | Acute peptic ulcer with hemorrhage and perforation | SNOMED | Condition | Standard | Yes | No | No |
| 27026 | 47064007 | Acute peptic ulcer with hemorrhage AND with perforation but without obstruction | SNOMED | Condition | Standard | Yes | No | No |
| 31335 | 22157005 | Acute peptic ulcer with hemorrhage but without obstruction | SNOMED | Condition | Standard | Yes | No | No |
| 194986 | 28945005 | Acute peptic ulcer with hemorrhage, with perforation AND with obstruction | SNOMED | Condition | Standard | Yes | No | No |
| 28779 | 17709002 | Bleeding esophageal varices | SNOMED | Condition | Standard | Yes | No | No |
| 26441 | 57748001 | Bleeding ulcer of esophagus | SNOMED | Condition | Standard | Yes | No | No |
| 4232181 | 89469000 | Chronic duodenal ulcer with hemorrhage | SNOMED | Condition | Standard | Yes | No | No |
| 437323 | 34021006 | Chronic duodenal ulcer with hemorrhage AND obstruction | SNOMED | Condition | Standard | Yes | No | No |
| 4289830 | 36975000 | Chronic duodenal ulcer with hemorrhage AND perforation | SNOMED | Condition | Standard | Yes | No | No |
| 438796 | 81142005 | Chronic duodenal ulcer with hemorrhage AND with perforation but without obstruction | SNOMED | Condition | Standard | Yes | No | No |
| 436148 | 62341002 | Chronic duodenal ulcer with hemorrhage but without obstruction | SNOMED | Condition | Standard | Yes | No | No |
| 440756 | 86258000 | Chronic duodenal ulcer with hemorrhage, with perforation AND with obstruction | SNOMED | Condition | Standard | Yes | No | No |
| 4211001 | 57246001 | Chronic gastric ulcer with hemorrhage | SNOMED | Condition | Standard | Yes | No | No |
| 201885 | 85859006 | Chronic gastric ulcer with hemorrhage and with obstruction | SNOMED | Condition | Standard | Yes | No | No |
| 4294973 | 76181002 | Chronic gastric ulcer with hemorrhage and with perforation | SNOMED | Condition | Standard | Yes | No | No |
| 196442 | 74341002 | Chronic gastric ulcer with hemorrhage AND with perforation but without obstruction | SNOMED | Condition | Standard | Yes | No | No |
| 197018 | 76078009 | Chronic gastric ulcer with hemorrhage but without obstruction | SNOMED | Condition | Standard | Yes | No | No |
| 198801 | 85787009 | Chronic gastric ulcer with hemorrhage, with perforation and with obstruction | SNOMED | Condition | Standard | Yes | No | No |
| 433515 | 62838000 | Chronic gastrojejunal ulcer with hemorrhage | SNOMED | Condition | Standard | Yes | No | No |
| 436729 | 90257004 | Chronic gastrojejunal ulcer with hemorrhage and obstruction | SNOMED | Condition | Standard | Yes | No | No |
| 4164920 | 45640006 | Chronic gastrojejunal ulcer with hemorrhage and perforation | SNOMED | Condition | Standard | Yes | No | No |
| 437326 | 46523000 | Chronic gastrojejunal ulcer with hemorrhage and with perforation but without obstruction | SNOMED | Condition | Standard | Yes | No | No |
| 443779 | 24001002 | Chronic gastrojejunal ulcer with hemorrhage, with perforation and with obstruction | SNOMED | Condition | Standard | Yes | No | No |
| 4174044 | 49232000 | Chronic peptic ulcer with hemorrhage | SNOMED | Condition | Standard | Yes | No | No |
| 24076 | 56461008 | Chronic peptic ulcer with hemorrhage AND obstruction | SNOMED | Condition | Standard | Yes | No | No |
| 4247008 | 61300005 | Chronic peptic ulcer with hemorrhage AND perforation | SNOMED | Condition | Standard | Yes | No | No |
| 22665 | 55746001 | Chronic peptic ulcer with hemorrhage AND with perforation but without obstruction | SNOMED | Condition | Standard | Yes | No | No |
| 30770 | 81518000 | Chronic peptic ulcer with hemorrhage but without obstruction | SNOMED | Condition | Standard | Yes | No | No |
| 24397 | 77661009 | Chronic peptic ulcer with hemorrhage, with perforation AND with obstruction | SNOMED | Condition | Standard | Yes | No | No |
| 198798 | 109558001 | Dieulafoy's vascular malformation | SNOMED | Condition | Standard | Yes | No | No |
| 4031954 | 23812009 | Duodenal ulcer with hemorrhage AND perforation | SNOMED | Condition | Standard | Yes | No | No |
| 23245 | 15238002 | Esophageal bleeding | SNOMED | Condition | Standard | Yes | No | No |
| 4112183 | 195475003 | Esophageal varices with bleeding, associated with another disorder | SNOMED | Condition | Standard | Yes | No | No |
| 193250 | 61401005 | Gastric hemorrhage | SNOMED | Condition | Standard | Yes | No | No |
| 45757783 | 40241000119109 | Gastric hemorrhage due to alcoholic gastritis | SNOMED | Condition | Standard | Yes | No | No |
| 46270145 | 150721000119102 | Gastric hemorrhage due to atrophic gastritis | SNOMED | Condition | Standard | Yes | No | No |
| 46270025 | 123411000119106 | Gastric hemorrhage due to eosinophilic gastritis | SNOMED | Condition | Standard | Yes | No | No |
| 46269911 | 1087161000119100 | Gastric hemorrhage due to hypertrophic gastritis | SNOMED | Condition | Standard | Yes | No | No |
| 192671 | 74474003 | Gastrointestinal hemorrhage | SNOMED | Condition | Standard | Yes | No | No |
| 26727 | 8765009 | Hematemesis | SNOMED | Condition | Standard | Yes | No | No |
| 443530 | 405729008 | Hematochezia | SNOMED | Condition | Standard | Yes | No | No |
| 442190 | 95540002 | Hemorrhage of colon | SNOMED | Condition | Standard | Yes | No | No |
| 45757543 | 190191000119107 | Hemorrhage of colon due to diverticulosis | SNOMED | Condition | Standard | Yes | No | No |
| 197925 | 266464001 | Hemorrhage of rectum and anus | SNOMED | Condition | Standard | Yes | No | No |
| 46269901 | 1086461000119100 | Hemorrhage of small intestine due to diverticulitis | SNOMED | Condition | Standard | Yes | No | No |
| 46270529 | 40271000119102 | Hemorrhage of small intestine with diverticulosis | SNOMED | Condition | Standard | Yes | No | No |
| 437027 | 95531001 | Hemorrhagic duodenitis | SNOMED | Condition | Standard | Yes | No | No |
| 46273183 | 712510007 | Intestinal hemorrhage | SNOMED | Condition | Standard | Yes | No | No |
| 45757654 | 29731000119103 | Intestinal hemorrhage due to angiodysplasia of intestine | SNOMED | Condition | Standard | Yes | No | No |
| 46269907 | 1086601000119100 | Intestinal hemorrhage with diverticulosis | SNOMED | Condition | Standard | Yes | No | No |
| 316457 | 35265002 | Mallory-Weiss syndrome | SNOMED | Condition | Standard | Yes | No | No |
| 4103703 | 2901004 | Melena | SNOMED | Condition | Standard | Yes | No | No |
| 4291649 | 37372002 | Upper gastrointestinal bleeding | SNOMED | Condition | Standard | Yes | No | No |
| 4226021 | 405538007 | Spontaneous hemorrhage | SNOMED | Condition | Standard | Yes | No | Yes |
| 433778 | 31056006 | Orbital hemorrhage | SNOMED | Condition | Standard | Yes | No | Yes |
| 4247597 | 93478000 | Intraocular hemorrhage | SNOMED | Condition | Standard | Yes | No | Yes |
| 439847 | 1386000 | Intracranial hemorrhage | SNOMED | Condition | Standard | Yes | No | Yes |
| 380113 | 14460007 | Hemorrhage in optic nerve sheaths | SNOMED | Condition | Standard | Yes | No | Yes |
| 258449 | 23412002 | Hemopericardium | SNOMED | Condition | Standard | Yes | No | Yes |
| 4299449 | 385494008 | Hematoma | SNOMED | Condition | Standard | Yes | No | Yes |
| 76784 | 81808003 | Hemarthrosis | SNOMED | Condition | Standard | Yes | No | Yes |
| 4308835 | 213262007 | Postoperative hematoma formation | SNOMED | Condition | Standard | No | Yes | Yes |
| 198221 | 5740008 | Pelvic hematoma during delivery | SNOMED | Condition | Standard | No | Yes | Yes |
| 4131053 | 264558008 | Pelvic hematoma | SNOMED | Condition | Standard | No | Yes | Yes |
| 376419 | 88050005 | Hematoma of pinna | SNOMED | Condition | Standard | No | Yes | Yes |
| 4169954 | 417941003 | Genitourinary tract hemorrhage | SNOMED | Condition | Standard | No | Yes | Yes |
| 378756 | 21117005 | Conjunctival hemorrhage | SNOMED | Condition | Standard | No | Yes | Yes |
| 4310674 | 8554002 | Brazilian purpuric fever | SNOMED | Condition | Standard | No | Yes | Yes |
| 4249574 | 72986009 | Acute hemorrhagic leukoencephalitis | SNOMED | Condition | Standard | No | Yes | Yes |
| 4161830 | 398264003 | Acute hemorrhagic conjunctivitis | SNOMED | Condition | Standard | No | Yes | Yes |
| 4343221 | 239186001 | Acute epidemic conjunctivitis | SNOMED | Condition | Standard | No | Yes | Yes |
| 138027 | 54288002 | Traumatic subcutaneous emphysema | SNOMED | Condition | Standard | No | Yes | Yes |
| 201478 | 64169002 | Traumatic shock | SNOMED | Condition | Standard | No | Yes | Yes |
| 440921 | 417746004 | Traumatic injury | SNOMED | Condition | Standard | No | Yes | Yes |
| 438658 | 32144005 | Traumatic anuria | SNOMED | Condition | Standard | No | Yes | Yes |
| 439235 | 276853009 | Self inflicted injury | SNOMED | Condition | Standard | No | Yes | Yes |
| 443272 | 269735005 | Injury undetermined whether accidentally or purposely inflicted | SNOMED | Condition | Standard | No | Yes | Yes |
| 434252 | 219256006 | Injury due to legal intervention | SNOMED | Condition | Standard | No | Yes | Yes |
| 442019 | 116224001 | Complication of procedure | SNOMED | Condition | Standard | No | Yes | Yes |
| 2720554 | P9056 | Whole blood, leukocytes reduced, irradiated, each unit | HCPCS | Device | Standard | Yes | No | Yes |
| 2720552 | P9054 | Whole blood or red blood cells, leukocytes reduced, frozen, deglycerol, washed, each unit | HCPCS | Device | Standard | Yes | No | Yes |
| 2720549 | P9051 | Whole blood or red blood cells, leukocytes reduced, cmv-negative, each unit | HCPCS | Device | Standard | Yes | No | Yes |
| 2720528 | P9022 | Red blood cells, washed, each unit | HCPCS | Device | Standard | Yes | No | Yes |
| 2720539 | P9040 | Red blood cells, leukocytes reduced, irradiated, each unit | HCPCS | Device | Standard | Yes | No | Yes |
| 2720522 | P9016 | Red blood cells, leukocytes reduced, each unit | HCPCS | Device | Standard | Yes | No | Yes |
| 2720556 | P9058 | Red blood cells, leukocytes reduced, cmv-negative, irradiated, each unit | HCPCS | Device | Standard | Yes | No | Yes |
| 2720537 | P9038 | Red blood cells, irradiated, each unit | HCPCS | Device | Standard | Yes | No | Yes |
| 2720555 | P9057 | Red blood cells, frozen/deglycerolized/washed, leukocytes reduced, irradiated, each unit | HCPCS | Device | Standard | Yes | No | Yes |
| 2720527 | P9021 | Red blood cells, each unit | HCPCS | Device | Standard | Yes | No | Yes |
| 2720538 | P9039 | Red blood cells, deglycerolized, each unit | HCPCS | Device | Standard | Yes | No | Yes |
| 915816 | P9072 | Platelets, pheresis, pathogen reduced or rapid bacterial tested, each unit | HCPCS | Device | Standard | Yes | No | Yes |
| 2720536 | P9037 | Platelets, pheresis, leukocytes reduced, irradiated, each unit | HCPCS | Device | Standard | Yes | No | Yes |
| 2720534 | P9035 | Platelets, pheresis, leukocytes reduced, each unit | HCPCS | Device | Standard | Yes | No | Yes |
| 2720551 | P9053 | Platelets, pheresis, leukocytes reduced, cmv-negative, irradiated, each unit | HCPCS | Device | Standard | Yes | No | Yes |
| 2720535 | P9036 | Platelets, pheresis, irradiated, each unit | HCPCS | Device | Standard | Yes | No | Yes |
| 2720533 | P9034 | Platelets, pheresis, each unit | HCPCS | Device | Standard | Yes | No | Yes |
| 2720532 | P9033 | Platelets, leukocytes reduced, irradiated, each unit | HCPCS | Device | Standard | Yes | No | Yes |
| 2720530 | P9031 | Platelets, leukocytes reduced, each unit | HCPCS | Device | Standard | Yes | No | Yes |
| 2720553 | P9055 | Platelets, leukocytes reduced, cmv-negative, apheresis/pheresis, each unit | HCPCS | Device | Standard | Yes | No | Yes |
| 2720531 | P9032 | Platelets, irradiated, each unit | HCPCS | Device | Standard | Yes | No | Yes |
| 2720550 | P9052 | Platelets, hla-matched leukocytes reduced, apheresis/pheresis, each unit | HCPCS | Device | Standard | Yes | No | Yes |
| 2720525 | P9019 | Platelets, each unit | HCPCS | Device | Standard | Yes | No | Yes |
| 2720526 | P9020 | Platelet rich plasma, each unit | HCPCS | Device | Standard | Yes | No | Yes |
| 2720529 | P9023 | Plasma, pooled multiple donor, solvent/detergent treated, frozen, each unit | HCPCS | Device | Standard | Yes | No | Yes |
| 915814 | P9070 | Plasma, pooled multiple donor, pathogen reduced, frozen, each unit | HCPCS | Device | Standard | Yes | No | Yes |
| 915815 | P9071 | Plasma (single donor), pathogen reduced, frozen, each unit | HCPCS | Device | Standard | Yes | No | Yes |
| 2720558 | P9060 | Fresh frozen plasma, donor retested, each unit | HCPCS | Device | Standard | Yes | No | Yes |
| 2720557 | P9059 | Fresh frozen plasma between 8-24 hours of collection, each unit | HCPCS | Device | Standard | Yes | No | Yes |
| 2720523 | P9017 | Fresh frozen plasma (single donor), frozen within 8 hours of collection, each unit | HCPCS | Device | Standard | Yes | No | Yes |
| 2720517 | P9011 | Blood, split unit | HCPCS | Device | Standard | Yes | No | Yes |
| 2720516 | P9010 | Blood (whole), for transfusion, per unit | HCPCS | Device | Standard | Yes | No | Yes |
| 2720543 | P9044 | Plasma, cryoprecipitate reduced, each unit | HCPCS | Drug | Standard | Yes | No | Yes |
| 35605208 | 1729441 | plasma protein fraction 50 MG/ML Injection | RxNorm | Drug | Standard | Yes | No | Yes |
| 2720518 | P9012 | Cryoprecipitate, each unit | HCPCS | Drug | Standard | Yes | No | Yes |
| 2108119 | 36430 | Transfusion, blood or blood components | CPT4 | Procedure | Standard | Yes | No | Yes |
| 2008236 | 99 | Transfusion of previously collected autologous blood | ICD9Proc | Procedure | Standard | Yes | No | Yes |
| 2008241 | 99 | Transfusion of other serum | ICD9Proc | Procedure | Standard | Yes | No | Yes |
| 4253788 | 74287006 | Transfusion of coagulation factors | SNOMED | Procedure | Standard | Yes | No | Yes |
| 4301111 | 78281001 | Transfusion of blood expander | SNOMED | Procedure | Standard | Yes | No | Yes |
| 4130829 | 12719002 | Platelet transfusion | SNOMED | Procedure | Standard | Yes | No | Yes |
| 4125928 | 288170000 | Packed blood cell transfusion | SNOMED | Procedure | Standard | Yes | No | Yes |
| 2008237 | 99 | Other transfusion of whole blood | ICD9Proc | Procedure | Standard | Yes | No | Yes |
| 2867454 | 3028 | Administration @ Circulatory @ Transfusion @ Vein | ICD10PCS | Procedure | Standard | Yes | No | Yes |
| 2820535 | 3023 | Administration @ Circulatory @ Transfusion @ Peripheral Vein | ICD10PCS | Procedure | Standard | Yes | No | Yes |
| 2880382 | 3024 | Administration @ Circulatory @ Transfusion @ Central Vein | ICD10PCS | Procedure | Standard | Yes | No | Yes |
| 2110570 | 62010 | Elevation of depressed skull fracture; with repair of dura and/or debridement of brain | CPT4 | Procedure | Standard | No | Yes | Yes |
| 2110568 | 62000 | Elevation of depressed skull fracture; simple, extradural | CPT4 | Procedure | Standard | No | Yes | Yes |
| 2110569 | 62005 | Elevation of depressed skull fracture; compound or comminuted, extradural | CPT4 | Procedure | Standard | No | Yes | Yes |
| 435713 | 274215009 | Transport accident | SNOMED | Observation | Standard | No | Yes | Yes |
| 4072126 | 219177001 | Homicide and assault by fight, brawl and rape | SNOMED | Observation | Standard | No | Yes | Yes |
| 4301487 | 77993003 | Exposure to attack by other person | SNOMED | Observation | Standard | No | Yes | Yes |
| 435991 | 217082002 | Accidental fall | SNOMED | Observation | Standard | No | Yes | Yes |
| 437167 | 218081007 | Accident caused by firearm missile | SNOMED | Observation | Standard | No | Yes | Yes |
| 4055518 | 242777003 | Accident caused by explosion | SNOMED | Observation | Standard | No | Yes | Yes |

**eTable 3: Features**

| **Features** | **Description/**  **Question** | **Data source** | **OMOP concept ID** | **Source concept code** | **Concept name** | **Vocabulary** | **Domain** | **Standard concept caption** | **Include** | **Exclude** | **Descendants** |
| --- | --- | --- | --- | --- | --- | --- | --- | --- | --- | --- | --- |
| Male | What was your biological sex assigned at birth? | Basics Survey | 45880669 | 1585846 | SexAtBirth_Male | PPI | Survey | Non-standard | Yes | No | No |
| Female |  | Basics Survey | 45878463 | 1585847 | SexAtBirth_Female | PPI | Survey | Non-standard | Yes | No | No |
| Age at index date | Calculated from birth date to index date | Basics Survey | - | - | date_of_birth | - | Person | - | - | - | - |
|  |  | EHR | - | - | date_of_first_drug_exposure | - | Drug | - | - | - | - |
| Hispanic | Which categories describe you? Select all that apply. | Basics Survey | 38003563 | Hispanic | Hispanic or Latino | PPI | Person | Standard | Yes | No | No |
| Asian |  | Basics Survey | 8515 | 2 | Asian | PPI | Person | Standard | Yes | No | No |
| Black or African American |  | Basics Survey | 8516 | 3 | Black or African American | PPI | Person | Standard | Yes | No | No |
| White |  | Basics Survey | 8527 | 5 | White | PPI | Person | Standard | Yes | No | No |
| No Highschool Degree | What is the highest grade or year of school you completed? | Basics Survey | 2000000007 | 2000000007 | HighestGrade_GeneralizedNoHighSchoolDegree | PPI | Survey | Non-standard | Yes | No | No |
| Highschool graduate |  | Basics Survey | 43021808 | 1585945 | HighestGrade_TwelveOrGED | SNOMED | Survey | Standard | Yes | No | No |
| College 1-3 years |  | Basics Survey | 4260980 | 1585946 | HighestGrade_CollegeOnetoThree | SNOMED | Survey | Standard | Yes | No | No |
| College 4 years or more or advanced degree |  | Basics Survey | 2000000006 | 2000000006 | HighestGrade_GeneralizedCollegeAndAbove | PPI | Survey | Non-standard | Yes | No | No |
| Employed for wages or self-employed | What is your current employment status? | Basics Survey | 2000000004 | 2000000004 | EmploymentStatus_GeneralizedEmployed | PPI | Survey | Non-standard | Yes | No | No |
| Less than $10,000 | What is your annual household income from all sources? | Basics Survey | 1585376 | 1585376 | AnnualIncome_less10k | PPI | Survey | Non-standard | Yes | No | No |
| $10,000- $24,999 |  | Basics Survey | 1585377 | 1585377 | AnnualIncome_10k25k | PPI | Survey | Non-standard | Yes | No | No |
| $25,000- $34,999 |  | Basics Survey | 1585378 | 1585378 | AnnualIncome_25k35k | PPI | Survey | Non-standard | Yes | No | No |
| $35,000- $49,999 |  | Basics Survey | 1585379 | 1585379 | AnnualIncome_35k50k | PPI | Survey | Non-standard | Yes | No | No |
| $50,000- $74,999 |  | Basics Survey | 1585380 | 1585380 | AnnualIncome_50k75k | PPI | Survey | Non-standard | Yes | No | No |
| $75,000-$99,999 |  | Basics Survey | 1585381 | 1585381 | AnnualIncome_75k100k | PPI | Survey | Non-standard | Yes | No | No |
| $100,000- $149,999 |  | Basics Survey | 1585382 | 1585382 | AnnualIncome_100k150k | PPI | Survey | Non-standard | Yes | No | No |
| $150,000- $199,999 |  | Basics Survey | 1585383 | 1585383 | AnnualIncome_150k200k | PPI | Survey | Non-standard | Yes | No | No |
| $200,000 or more |  | Basics Survey | 1585384 | 1585384 | AnnualIncome_more200k | PPI | Survey | Non-standard | Yes | No | No |
| Health insurance | Are you covered by health insurance or some other kind of health care plan? | Basics Survey | 45877994 | 1585387 | HealthInsurance_Yes | LOINC | Survey | Standard | Yes | No | No |
| Brief health literacy screen^a^ | How confident are you filling out medical forms by yourself? | Overall Health Survey | 1585766 | 1585766 | OverallHealth_MedicalFormConfidence | PPI | Survey | Non-standard | Yes | No | No |
|  | How often do you have someone help you read health-related materials? | Overall Health Survey | 1585772 | 1585772 | OverallHealth_HealthMaterialAssistance | PPI | Survey | Non-standard | Yes | No | No |
|  | How often do you have problems learning about your medical condition because of difficulty understanding written information? | Overall Health Survey | 1585778 | 1585778 | OverallHealth_DifficultUnderstandInfo | PPI | Survey | Non-standard | Yes | No | No |
| Hypertension | Diagnosis of hypertension (prior to index date) | EHR | 316866 | 38341003 | Hypertensive disorder | SNOMED | Condition | Standard | Yes | No | Yes |
|  |  | EHR | 42709887 | 449759005 | Hypertensive complication | SNOMED | Condition | Standard | Yes | No | Yes |
|  |  | EHR | 4279525 | 367390009 | Hypertension in the obstetric context | SNOMED | Condition | Standard | No | Yes | Yes |
|  |  | EHR | 4071202 | 206596003 | Neonatal hypertension | SNOMED | Condition | Standard | No | Yes | Yes |
|  |  | EHR | 4167493 | 48194001 | Pregnancy-induced hypertension | SNOMED | Condition | Standard | No | Yes | Yes |
|  |  | PMH Survey | 4058286 | 43529063 | Has a doctor or health care provider ever told you that you have...Hypertension? | PPI | Survey | Non-standard | Yes | No | No |
| Renal disease | Diagnosis of renal disease (prior to index date) | EHR | 198124 | 90708001 | Kidney disease | SNOMED | Condition | Standard | Yes | No | Yes |
|  |  | EHR | 440516 | 373585009 | Congenital ureterovesical obstruction | SNOMED | Condition | Standard | Yes | No | Yes |
|  |  | EHR | 133810 | 19034001 | Hyperparathyroidism due to renal insufficiency | SNOMED | Condition | Standard | Yes | No | Yes |
|  |  | EHR | 437992 | 167180005 | Renal function tests abnormal | SNOMED | Condition | Standard | Yes | No | Yes |
|  |  | EHR | 197921 | 16726004 | Renal osteodystrophy | SNOMED | Condition | Standard | Yes | No | Yes |
|  |  | EHR | 440828 | 204967008 | Renal pelvis and ureter obstructive defects | SNOMED | Condition | Standard | Yes | No | Yes |
|  |  | EHR | 199870 | 16934004 | Renal vascular disorder | SNOMED | Condition | Standard | Yes | No | Yes |
|  |  | EHR | 192440 | 12818004 | Ureterocele | SNOMED | Condition | Standard | Yes | No | Yes |
|  |  | PMH Survey | 43529136 | Cancer Conditions: Kidney Cancer | Has a doctor or health care provider ever told you that you have…Kidney Cancer? | PPI | Survey | Non-standard | Yes | No | No |
|  |  | PMH Survey | 1384395 | Kidney Conditions: Acute Kidney No Dialysis | Has a doctor or health care provider ever told you that you have…Acute Kidney No Dialysis? | PPI | Survey | Non-standard | Yes | No | No |
|  |  | PMH Survey | 1384563 | Kidney Conditions: Kidney With Dialysis | Has a doctor or health care provider ever told you that you have…Kidney With Dialysis? | PPI | Survey | Non-standard | Yes | No | No |
|  |  | PMH Survey | 1384414 | Kidney Conditions: Kidney Without Dialysis | Has a doctor or health care provider ever told you that you have…Kidney Without Dialysis? | PPI | Survey | Non-standard | Yes | No | No |
|  |  | PMH Survey | 1384515 | Kidney Conditions: Kidney Stones | Has a doctor or health care provider ever told you that you have…Kidney Stones? | PPI | Survey | Non-standard | Yes | No | No |
|  |  | PMH Survey | 1384646 | Kidney Conditions: Other Kidney Condition | Has a doctor or health care provider ever told you that you have…Other Kidney Condition? | PPI | Survey | Non-standard | Yes | No | No |
| Liver disease | Diagnosis of liver disease (prior to index date) | EHR | 194984 | 235856003 | Disease of liver | SNOMED | Condition | Standard | Yes | No | Yes |
|  |  | PMH Survey | 1384583 | Digestive System Conditions: Liver Condition | Has a doctor or health care provider ever told you that you have…Liver Condition (select all that apply)? | PPI | Survey | Non-standard | Yes | No | Yes |
|  |  | PMH Survey | 1384581 | Infectious Disease Condition: Hepatitis A | Has a doctor or health care provider ever told you that you have…Hepatitis A? | PPI | Survey | Non-standard | Yes | No | No |
|  |  | PMH Survey | 1384429 | Infectious Disease Condition: Hepatitis B | Has a doctor or health care provider ever told you that you have…Hepatitis B? | PPI | Survey | Non-standard | Yes | No | No |
|  |  | PMH Survey | 1384588 | Infectious Disease Condition: Hepatitis C | Has a doctor or health care provider ever told you that you have…Hepatitis C? | PPI | Survey | Non-standard | Yes | No | No |
| Cerebrovascular disease | Diagnosis of cerebrovascular disease (prior to index date) | EHR | 381591 | 62914000 | Cerebrovascular disease | SNOMED | Condition | Standard | Yes | No | Yes |
|  |  | EHR | 443454 | 432504007 | Cerebral infarction | SNOMED | Condition | Standard | Yes | No | Yes |
|  |  | EHR | 4353709 | 250845006 | Intracerebral vascular finding | SNOMED | Condition | Standard | Yes | No | Yes |
|  |  | EHR | 439847 | 1386000 | Intracranial hemorrhage | SNOMED | Condition | Standard | Yes | No | Yes |
|  |  | EHR | 376714 | 195199008 | Vertebrobasilar artery syndrome | SNOMED | Condition | Standard | Yes | No | Yes |
|  |  | PMH Survey | 43529874 | Circulatory Conditions: Stroke | Has a doctor or health care provider ever told you that you have…Stroke? | PPI | Survey | Non-standard | Yes | No | No |
|  |  | PMH Survey | 43529918 | Circulatory Conditions: Transient Ischemic Attack | Has a doctor or health care provider ever told you that you have…Transient Ischemic Attack? | PPI | Survey | Non-standard | Yes | No | No |
| Bleeding disorder | Diagnosis of bleeding disorder (prior to index date) | EHR | 4179872 | 362970003 | Disorder of hemostatic system | SNOMED | Condition | Standard | Yes | No | Yes |
|  |  | PMH Survey | 1384512 | Circulatory Conditions: Bleeding Disorder | Has a doctor or health care provider ever told you that you have…Bleeding Disorder? | PPI | Survey | Non-standard | Yes | No | No |
| Organ transplant | Have you had a transplant of any type? | PMH Survey | 1585804 | 45877994 | OrganTransplant_Yes | LOINC | Survey | Standard | Yes | No | No |
| Bleeding history | Past history of bleeding (prior to index date; see S2 for bleeding algorithm) | - | - | - | - | - | - | - | - | - | - |
| Alcohol use | In your entire life, have you had at least 1 drink of any kind of alcohol, not counting small tastes or sips? (By a “drink,” we mean a can or bottle of beer, a glass of wine or a wine cooler, a shot of liquor, or a mixed drink with liquor in it.) | Lifestyle Survey | 45877994 | 1586199 | AlcoholParticipant_Yes | LOINC | Survey | Standard | Yes | No | No |
| 100 cigarettes lifetime | Have you smoked at least 100 cigarettes in your entire life? (There are 20 cigarettes in a pack.) | Lifestyle Survey | 45877994 | 1585858 | 100CigsLifetime_Yes | LOINC | Survey | Standard | Yes | No | No |
| Cigar smoking | Have you ever smoked a traditional cigar, cigarillo, or filtered cigar, even one or two puffs? | Lifestyle Survey | 45877994 | 1586175 | CigarSmokeParticipant_Yes | PPI | Survey | Non-standard | Yes | No | No |
| Electronic smoking | Have you ever used an electronic nicotine product, even one or two times? (Electronic nicotine products include e-cigarettes, vape pens, hookah pens, personal vaporizers and mods, e-cigars, e-pipes, and e-hookahs.) | Lifestyle Survey | 45877994 | 1586167 | ElectricSmokeParticipant_Yes | LOINC | Survey | Standard | Yes | No | No |
| Hookah smoking | Have you ever smoked tobacco in a hookah, even one or two puffs? | Lifestyle Survey | 45877994 | 1586183 | HookahSmokeParticipant_Yes | LOINC | Survey | Standard | Yes | No | No |
| Smokeless tobacco | Have you ever used smokeless tobacco products, even one or two times? (Smokeless tobacco products include snus pouches, Skoal Bandits, loose snus, moist snuff, dip, spit, and chewing tobacco.) | Lifestyle Survey | 45877994 | 1586191 | SmokelessTobaccoParticipant_Yes | LOINC | Survey | Standard | Yes | No | No |
| Cocaine | In your LIFETIME, which of the following substances have you ever used?...Cocaine (coke, crack, etc.) | Lifestyle Survey | 45876771 | 1585638 | WhichDrugsUsed_CocaineUse | LOINC | Survey | Standard | Yes | No | No |
| Hallucinogens | In your LIFETIME, which of the following substances have you ever used?...Hallucinogens (LSD, acid, mushrooms, PCP, Special K, ecstasy, etc.) | Lifestyle Survey | 45881354 | 1585643 | WhichDrugsUsed_HallucinogensUse | LOINC | Survey | Standard | Yes | No | No |
| Inhalants | In your LIFETIME, which of the following substances have you ever used?...Inhalants (nitrous oxide, glue, gas, paint thinner, etc.) | Lifestyle Survey | 45883389 | 1585641 | WhichDrugsUsed_InhalantsUse | LOINC | Survey | Standard | Yes | No | No |
| Marijuana | In your LIFETIME, which of the following substances have you ever used?...Marijuana (cannabis, pot, grass, hash, weed, etc.) | Lifestyle Survey | 45881688 | 1585637 | WhichDrugsUsed_MarijuanaUse | LOINC | Survey | Standard | Yes | No | No |
| Methamphetamine | In your LIFETIME, which of the following substances have you ever used?...Other stimulants (methamphetamine, speed, crystal meth, ice, k2/spice, bath salts, etc.) | Lifestyle Survey | 1585640 | 1585640 | WhichDrugsUsed_MethamphetamineUse | PPI | Survey | Non-standard | Yes | No | No |
| Prescription opioids | In your LIFETIME, which of the following substances have you ever used?...Prescription opioids for non-medical reasons (fentanyl, oxycodone [OxyContin, Percocet], hydrocodone [Vicodin], methadone, buprenorphine, etc.) | Lifestyle Survey | 1585645 | 1585645 | WhichDrugsUsed_PrescriptionOpioidsUse | PPI | Survey | Non-standard | Yes | No | No |
| Stimulants | In your LIFETIME, which of the following substances have you ever used? | Lifestyle Survey | 1585639 | 1585639 | WhichDrugsUsed_PrescriptionStimulantsUse | PPI | Survey | Non-standard | Yes | No | No |
| Sedatives | In your LIFETIME, which of the following substances have you ever used?...Sedatives or sleeping pills for non-medical reasons (Valium, Serepax, Ativan, Xanax, Librium, Rohypnol, GHB, etc.) | Lifestyle Survey | 45883390 | 1585642 | WhichDrugsUsed_SedativesUse | LOINC | Survey | Standard | Yes | No | No |
| Street opioids | In your LIFETIME, which of the following substances have you ever used?...Street opioids (heroin, opium, etc.) | Lifestyle Survey | 1585644 | 1585644 | WhichDrugsUsed_StreetOpioidsUse | PPI | Survey | Non-standard | Yes | No | No |
| Clopidogrel | Concurrent drug use holds the value between 0 and 1, where 0 indicates no overlap in drug use while 1 indicates 100% overlap in drug use between drug features and researched drugs during the follow-up period. | EHR | 1322184 | 32968 | clopidogrel | RxNorm | Drug | Standard | Yes | No | Yes |
| Warfarin |  | EHR | 1310149 | 11289 | warfarin | RxNorm | Drug | Standard | Yes | No | Yes |
| Apixaban |  | EHR | 43013024 | 1364430 | apixaban | RxNorm | Drug | Standard | Yes | No | Yes |
| Rivaroxaban |  | EHR | 40241331 | 1114195 | rivaroxaban | RxNorm | Drug | Standard | Yes | No | Yes |
| Dabigatran |  | EHR | 45775372 | 1546356 | dabigatran | RxNorm | Drug | Standard | Yes | No | Yes |
| Edoxaban |  | EHR | 45892847 | 1599538 | edoxaban | RxNorm | Drug | Standard | Yes | No | Yes |
| Ticagrelor |  | EHR | 40241186 | 1116632 | ticagrelor | RxNorm | Drug | Standard | Yes | No | Yes |
| Prasugrel |  | EHR | 40163718 | 613391 | prasugrel | RxNorm | Drug | Standard | Yes | No | Yes |
| Dipyridamole |  | EHR | 1331270 | 3521 | dipyridamole | RxNorm | Drug | Standard | Yes | No | Yes |
| Ticlopidine |  | EHR | 1302398 | 10594 | ticlopidine | RxNorm | Drug | Standard | Yes | No | Yes |
| Eptifibatide |  | EHR | 1322199 | 75635 | eptifibatide | RxNorm | Drug | Standard | Yes | No | Yes |
| Aspirin (low dose, <= 325mg/day) |  | EHR | 1112807 | 1191 | aspirin | RxNorm | Drug | Standard | Yes | No | Yes |
| Aspirin (high dose, >325 mg/day) |  | EHR |  |  |  |  |  |  |  |  |  |
| Ibuprofen |  | EHR | 1177480 | 5640 | ibuprofen | RxNorm | Drug | Standard | Yes | No | Yes |
| Indomethacin |  | EHR | 1178663 | 5781 | indomethacin | RxNorm | Drug | Standard | Yes | No | Yes |
| Naproxen |  | EHR | 1115008 | 7258 | naproxen | RxNorm | Drug | Standard | Yes | No | Yes |
| Mefenamic acid |  | EHR | 1197736 | 257844 | mefenamate | RxNorm | Drug | Standard | Yes | No | Yes |
| Ketorolac |  | EHR | 1136980 | 35827 | ketorolac | RxNorm | Drug | Standard | Yes | No | Yes |
| Meloxicam |  | EHR | 1150345 | 41493 | meloxicam | RxNorm | Drug | Standard | Yes | No | Yes |
| Celecoxib |  | EHR | 1118084 | 140587 | celecoxib | RxNorm | Drug | Standard | Yes | No | Yes |
| Diclofenac |  | EHR | 1124300 | 3355 | diclofenac | RxNorm | Drug | Standard | Yes | No | Yes |
|  |  | EHR | 4231622 | 359540000 | Topical | SNOMED | Drug (route) | Standard | No | Yes | Yes |
|  |  | EHR | 4263689 | 6064005 | Topical route | SNOMED | Drug (route) | Standard | No | Yes | Yes |
| Prednisone |  | EHR | 1551099 | 8640 | prednisone | RxNorm | Drug | Standard | Yes | No | Yes |
|  |  | EHR | 4132161 | 26643006 | Oral route | SNOMED | Drug (route) | Standard | Yes | No | Yes |
|  |  | EHR | 4142048 | 34206005 | Subcutaneous route | SNOMED | Drug (route) | Standard | Yes | No | Yes |
|  |  | EHR | 4302612 | 78421000 | Intramuscular route | SNOMED | Drug (route) | Standard | Yes | No | Yes |
|  |  | EHR | 4217202 | 72607000 | Intrathecal route | SNOMED | Drug (route) | Standard | Yes | No | Yes |
|  |  | EHR | 4157761 | 372475000 | Perineural route | SNOMED | Drug (route) | Standard | Yes | No | Yes |
|  |  | EHR | 4225555 | 404820008 | Epidural route | SNOMED | Drug (route) | Standard | Yes | No | Yes |
|  |  | EHR | 4112421 | 255560000 | Intravenous | SNOMED | Drug (route) | Standard | Yes | No | Yes |
|  |  | EHR | 4128794 | 260548002 | Oral | SNOMED | Drug (route) | Standard | Yes | No | Yes |
| Prednisolone |  | EHR | 1550557 | 8638 | prednisolone | RxNorm | Drug | Standard | Yes | No | Yes |
|  |  | EHR | 4132161 | 26643006 | Oral route | SNOMED | Drug (route) | Standard | Yes | No | Yes |
|  |  | EHR | 4142048 | 34206005 | Subcutaneous route | SNOMED | Drug (route) | Standard | Yes | No | Yes |
|  |  | EHR | 4302612 | 78421000 | Intramuscular route | SNOMED | Drug (route) | Standard | Yes | No | Yes |
|  |  | EHR | 4217202 | 72607000 | Intrathecal route | SNOMED | Drug (route) | Standard | Yes | No | Yes |
|  |  | EHR | 4157761 | 372475000 | Perineural route | SNOMED | Drug (route) | Standard | Yes | No | Yes |
|  |  | EHR | 4225555 | 404820008 | Epidural route | SNOMED | Drug (route) | Standard | Yes | No | Yes |
|  |  | EHR | 4112421 | 255560000 | Intravenous | SNOMED | Drug (route) | Standard | Yes | No | Yes |
|  |  | EHR | 4128794 | 260548002 | Oral | SNOMED | Drug (route) | Standard | Yes | No | Yes |
| Methylprednisolone |  | EHR | 1506270 | 6902 | methylprednisolone | RxNorm | Drug | Standard | Yes | No | Yes |
|  |  | EHR | 4132161 | 26643006 | Oral route | SNOMED | Drug (route) | Standard | Yes | No | Yes |
|  |  | EHR | 4142048 | 34206005 | Subcutaneous route | SNOMED | Drug (route) | Standard | Yes | No | Yes |
|  |  | EHR | 4302612 | 78421000 | Intramuscular route | SNOMED | Drug (route) | Standard | Yes | No | Yes |
|  |  | EHR | 4217202 | 72607000 | Intrathecal route | SNOMED | Drug (route) | Standard | Yes | No | Yes |
|  |  | EHR | 4157761 | 372475000 | Perineural route | SNOMED | Drug (route) | Standard | Yes | No | Yes |
|  |  | EHR | 4225555 | 404820008 | Epidural route | SNOMED | Drug (route) | Standard | Yes | No | Yes |
|  |  | EHR | 4112421 | 255560000 | Intravenous | SNOMED | Drug (route) | Standard | Yes | No | Yes |
|  |  | EHR | 4128794 | 260548002 | Oral | SNOMED | Drug (route) | Standard | Yes | No | Yes |
| Dexamethasone |  | EHR | 1518254 | 3264 | dexamethasone | RxNorm | Drug | Standard | Yes | No | Yes |
|  |  | EHR | 4132161 | 26643006 | Oral route | SNOMED | Drug (route) | Standard | Yes | No | Yes |
|  |  | EHR | 4142048 | 34206005 | Subcutaneous route | SNOMED | Drug (route) | Standard | Yes | No | Yes |
|  |  | EHR | 4302612 | 78421000 | Intramuscular route | SNOMED | Drug (route) | Standard | Yes | No | Yes |
|  |  | EHR | 4217202 | 72607000 | Intrathecal route | SNOMED | Drug (route) | Standard | Yes | No | Yes |
|  |  | EHR | 4157761 | 372475000 | Perineural route | SNOMED | Drug (route) | Standard | Yes | No | Yes |
|  |  | EHR | 4225555 | 404820008 | Epidural route | SNOMED | Drug (route) | Standard | Yes | No | Yes |
|  |  | EHR | 4112421 | 255560000 | Intravenous | SNOMED | Drug (route) | Standard | Yes | No | Yes |
|  |  | EHR | 4128794 | 260548002 | Oral | SNOMED | Drug (route) | Standard | Yes | No | Yes |
| Hydrocortisone |  | EHR | 975125 | 5492 | hydrocortisone | RxNorm | Drug | Standard | Yes | No | Yes |
|  |  | EHR | 4132161 | 26643006 | Oral route | SNOMED | Drug (route) | Standard | Yes | No | Yes |
|  |  | EHR | 4142048 | 34206005 | Subcutaneous route | SNOMED | Drug (route) | Standard | Yes | No | Yes |
|  |  | EHR | 4302612 | 78421000 | Intramuscular route | SNOMED | Drug (route) | Standard | Yes | No | Yes |
|  |  | EHR | 4217202 | 72607000 | Intrathecal route | SNOMED | Drug (route) | Standard | Yes | No | Yes |
|  |  | EHR | 4157761 | 372475000 | Perineural route | SNOMED | Drug (route) | Standard | Yes | No | Yes |
|  |  | EHR | 4225555 | 404820008 | Epidural route | SNOMED | Drug (route) | Standard | Yes | No | Yes |
|  |  | EHR | 4112421 | 255560000 | Intravenous | SNOMED | Drug (route) | Standard | Yes | No | Yes |
|  |  | EHR | 4128794 | 260548002 | Oral | SNOMED | Drug (route) | Standard | Yes | No | Yes |
| Citalopram | Current SSRI use^b^ | EHR | 797617 | 2556 | citalopram | RxNorm | Drug | Standard | Yes | No | Yes |
| Escitalopram |  | EHR | 715939 | 321988 | escitalopram | RxNorm | Drug | Standard | Yes | No | Yes |
| Fluoxetine |  | EHR | 755695 | 4493 | fluoxetine | RxNorm | Drug | Standard | Yes | No | Yes |
| Fluvoxamine |  | EHR | 751412 | 42355 | fluvoxamine | RxNorm | Drug | Standard | Yes | No | Yes |
| Paroxetine |  | EHR | 722031 | 32937 | paroxetine | RxNorm | Drug | Standard | Yes | No | Yes |
| Sertraline |  | EHR | 739138 | 36437 | sertraline | RxNorm | Drug | Standard | Yes | No | Yes |
| Vortioxetine |  | EHR | 44507700 | 1455099 | vortioxetine | RxNorm | Drug | Standard | Yes | No | Yes |
| None | SSRI used just before newly prescribed SSRI^b^ | EHR | - | - | - | - | - | - | - | - | - |
| Citalopram |  | EHR | 797617 | 2556 | citalopram | RxNorm | Drug | Standard | Yes | No | Yes |
| Escitalopram |  | EHR | 715939 | 321988 | escitalopram | RxNorm | Drug | Standard | Yes | No | Yes |
| Fluoxetine |  | EHR | 755695 | 4493 | fluoxetine | RxNorm | Drug | Standard | Yes | No | Yes |
| Fluvoxamine |  | EHR | 751412 | 42355 | fluvoxamine | RxNorm | Drug | Standard | Yes | No | Yes |
| Paroxetine |  | EHR | 722031 | 32937 | paroxetine | RxNorm | Drug | Standard | Yes | No | Yes |
| Sertraline |  | EHR | 739138 | 36437 | sertraline | RxNorm | Drug | Standard | Yes | No | Yes |
| Vortioxetine |  | EHR | 44507700 | 1455099 | vortioxetine | RxNorm | Drug | Standard | Yes | No | Yes |
| Number of prior SSRI switches | Number of prior SSRI switches (continuous variable)^b^ | EHR | - | - | - | - | - | - | - | - | - |

^a^ Responses were assigned a number from 1 to 5 and then summed. Higher scores reflecting worse self-reported health literacy (range: 3-15). Leave blank if missing 1 or more responses.

^b^ Features found only in the combined SSRI dataset.

**eTable 4: Hyperparameters of best models**

| **Cohorts** | **Best Model AUC Score** | **Model** | **Hyperparameters** |
| --- | --- | --- | --- |
| Clopidogrel | 0.63791809 | Logisitc Regression | penalty: 'l2', solver: 'liblinear', maxiter: 1000, C: 0.1, random_state: 42 |
| Warfarin | 0.68181479 | XGBoost | max_depth: 2, min_child_weight: 1, gamma: 0.4, reg_alpha: 0.01, reg_lambda: 1, random_state: 42 |
| Escitalopram | 0.65611523 | Random Forest | max_depth: 1, n_estimators: 700, random_state: 42 |
| Citalopram | 0.6975242 | Random Forest | max_depth: 3, n_estimators: 700, random_state: 42 |
| Fluvoxamine | 0.77090909 | XGBoost | max_depth: 1, min_child_weight: 2, gamma: 0.1, reg_alpha: 0.01, reg_lambda: 1, random_state: 42 |
| Fluoxetine | 0.66378586 | Decision Tree | max_depth: 7, min_samples_split: 8, min_samples_leaf: 1, max_features: 'log2', random_state: 42 |
| Sertraline | 0.66537992 | Random Forest | max_depth: 9, n_estimators: 700, random_state: 42 |
| Vortioxetine | 0.80164835 | Logisitc Regression | penalty: 'l2', solver: 'liblinear', maxiter: 1000, C: 1, random_state: 42 |
| Paroxetine | 0.63226865 | Random Forest | max_depth: 2, n_estimators: 300, random_state: 42 |
| Combined SSRI | 0.68756549 | XGBoost | max_depth: 3, min_child_weight: 5, gamma: 0.1, reg_alpha: 1, reg_lambda: 0.01, random_state: 42 |

Abbreviations: AUC - area under the receiver operating characteristic curve statistic.

**eTable 5: AUC score for all models**

| **Cohorts** | **Logistic Regression** | | | | **Decision tree** | | | | **Random Forest** | | | | **XGBoost** | | | |
| --- | --- | --- | --- | --- | --- | --- | --- | --- | --- | --- | --- | --- | --- | --- | --- | --- |
|  | **Without Feature Selection** | | **With Feature Selection** | | **Without Feature Selection** | | **With Feature Selection** | | **Without Feature Selection** | | **With Feature Selection** | | **Without Feature Selection** | | **With Feature Selection** | |
|  | **AUC** | **UM AUC** | **AUC** | **UM AUC** | **AUC** | **UM AUC** | **AUC** | **UM AUC** | **AUC** | **UM AUC** | **AUC** | **UM AUC** | **AUC** | **UM AUC** | **AUC** | **UM AUC** |
| Clopidogrel | 0.63791809 | 0.63400931 | 0.63791809 | 0.63396508 | 0.61441945 | 0.62460192 | 0.61610149 | 0.60894679 | 0.63594171 | 0.63560274 | 0.63148687 | 0.63223624 | 0.63737683 | 0.61998778 | 0.63756427 | 0.62448238 |
| Warfarin | 0.6643433 | 0.6625464 | 0.6643433 | 0.6625464 | 0.64314336 | 0.63505228 | 0.64976167 | 0.64386808 | 0.67526169 | 0.68339923 | 0.67670603 | 0.68361657 | 0.68181479 | 0.67932546 | 0.68313733 | 0.67662388 |
| Escitalopram | 0.64680432 | 0.63777155 | 0.64676496 | 0.63815225 | 0.59906755 | 0.61522569 | 0.61298884 | 0.60781852 | 0.65611523 | 0.6486792 | 0.65451887 | 0.65171263 | 0.65576711 | 0.63283817 | 0.65925759 | 0.63453832 |
| Citalopram | 0.67451979 | 0.677031 | 0.67575963 | 0.67805177 | 0.66774979 | 0.64771257 | 0.66941885 | 0.66612626 | 0.6975242 | 0.69473671 | 0.68675142 | 0.68393046 | 0.68631723 | 0.68675182 | 0.67474503 | 0.6846705 |
| Fluvoxamine | 0.56863636 | 0.57863636 | 0.56363636 | 0.57863636 | 0.76136364 | 0.67272727 | 0.76136364 | 0.67272727 | 0.70681818 | 0.59409091 | 0.72363636 | 0.64045455 | 0.77090909 | 0.71863636 | 0.77181818 | 0.74272727 |
| Fluoxetine | 0.64826555 | 0.65364312 | 0.64801064 | 0.65367804 | 0.66378586 | 0.63527202 | 0.64758537 | 0.62306147 | 0.65586572 | 0.64952887 | 0.65680807 | 0.64936864 | 0.65228374 | 0.64895979 | 0.65024445 | 0.64988267 |
| Sertraline | 0.62712726 | 0.6217837 | 0.62709815 | 0.62178379 | 0.60619977 | 0.60207113 | 0.61680838 | 0.61791254 | 0.66537992 | 0.66138389 | 0.6662658 | 0.65947706 | 0.65838701 | 0.6537071 | 0.65873742 | 0.65407645 |
| Vortioxetine | 0.80164835 | 0.8043956 | 0.80164835 | 0.8043956 | 0.66016484 | 0.78681319 | 0.74505495 | 0.68901099 | 0.73131868 | 0.73791209 | 0.72637363 | 0.70879121 | 0.72582418 | 0.74010989 | 0.73296703 | 0.74615385 |
| Paroxetine | 0.63001826 | 0.63414818 | 0.62960825 | 0.63414818 | 0.61382288 | 0.61382288 | 0.6424088 | 0.61840242 | 0.63226865 | 0.60680616 | 0.63491914 | 0.59625556 | 0.6186077 | 0.57297519 | 0.62241188 | 0.5641 |
| Combined SSRI | 0.66679108 | 0.66879001 | 0.66671508 | 0.66881219 | 0.65576526 | 0.63875399 | 0.65593691 | 0.63875399 | 0.68159227 | 0.67980741 | 0.68278661 | 0.68024354 | 0.68756549 | 0.6789606 | 0.6867541 | 0.6783384 |

Abbreviations: AUC - area under the receiver operating characteristic curve statistic; UM – upsampled minority.

**eTable 6: Clopidogrel performance statistics**

| **Youden index optimized parameters** | **Logistic Regression** | | | | **Decision tree** | | | | **Random Forest** | | | | **XGBoost** | | | |
| --- | --- | --- | --- | --- | --- | --- | --- | --- | --- | --- | --- | --- | --- | --- | --- | --- |
|  | **Without Feature Selection** | | **With Feature Selection** | | **Without Feature Selection** | | **With Feature Selection** | | **Without Feature Selection** | | **With Feature Selection** | | **Without Feature Selection** | | **With Feature Selection** | |
|  | **Score** | **UM Score** | **Score** | **UM Score** | **Score** | **UM Score** | **Score** | **UM Score** | **Score** | **UM Score** | **Score** | **UM Score** | **Score** | **UM Score** | **Score** | **UM Score** |
| Accuracy | 0.60082903 | 0.60911714 | 0.60082903 | 0.60911714 | 0.78880706 | 0.75919466 | 0.73785745 | 0.77717485 | 0.60543066 | 0.6776938 | 0.60356804 | 0.66461886 | 0.59944014 | 0.66097545 | 0.65445521 | 0.57198536 |
| Misclassification | 0.39917097 | 0.39088286 | 0.39917097 | 0.39088286 | 0.21119294 | 0.24080534 | 0.26214255 | 0.22282515 | 0.39456934 | 0.3223062 | 0.39643196 | 0.33538114 | 0.40055986 | 0.33902455 | 0.34554479 | 0.42801464 |
| Sensitivity | 0.64492754 | 0.65652174 | 0.64492754 | 0.65652174 | 0.32880435 | 0.37898551 | 0.44221014 | 0.33822464 | 0.65742754 | 0.57137681 | 0.66213768 | 0.56775362 | 0.66322464 | 0.52554348 | 0.59710145 | 0.66630435 |
| Specificity | 0.59535298 | 0.60296038 | 0.59535298 | 0.60296038 | 0.84464594 | 0.80516516 | 0.77335924 | 0.8306644 | 0.59905548 | 0.69032545 | 0.59647291 | 0.67623327 | 0.59190145 | 0.67743146 | 0.66116958 | 0.56037079 |
| Positive Predictive Value | 0.20170781 | 0.17884456 | 0.20170781 | 0.17884456 | 0.20434857 | 0.19327333 | 0.21943481 | 0.18047507 | 0.16777555 | 0.19583383 | 0.16903626 | 0.18537516 | 0.17152 | 0.18626782 | 0.18625366 | 0.17593005 |
| Negative Predictive Value | 0.9357544 | 0.93561078 | 0.9357544 | 0.93561078 | 0.91298962 | 0.91510834 | 0.92186665 | 0.91245248 | 0.93776965 | 0.93102421 | 0.93796034 | 0.92898747 | 0.93693758 | 0.92361893 | 0.93331876 | 0.93490264 |
| Positive Likelihood Ratio | 0.00648371 | 0.00660308 | 0.00648371 | 0.00660308 | 0.00331482 | 0.00381966 | 0.0044541 | 0.00340982 | 0.00661263 | 0.00575186 | 0.0066599 | 0.00571504 | 0.00667049 | 0.00528892 | 0.00600896 | 0.00669791 |
| Negative Likelihood Ratio | 204.409032 | 192.277216 | 204.409032 | 192.277216 | 119.25039 | 124.973076 | 133.10243 | 120.721674 | 170.088294 | 150.021822 | 171.975009 | 150.70786 | 175.693402 | 155.39602 | 154.344165 | 199.972909 |
| F1 Score | 0.27172817 | 0.27442938 | 0.27172817 | 0.27442938 | NA | 0.24540698 | 0.26429174 | NA | 0.26452808 | 0.28228671 | 0.26656792 | 0.27194766 | 0.26783428 | 0.25841303 | 0.27250135 | 0.2652947 |

Abbreviations: UM – upsampled minority.

**eTable 7: Warfarin performance statistics**

| **Youden index optimized parameters** | **Logistic Regression** | | | | **Decision tree** | | | | **Random Forest** | | | | **XGBoost** | | | |
| --- | --- | --- | --- | --- | --- | --- | --- | --- | --- | --- | --- | --- | --- | --- | --- | --- |
|  | **Without Feature Selection** | | **With Feature Selection** | | **Without Feature Selection** | | **With Feature Selection** | | **Without Feature Selection** | | **With Feature Selection** | | **Without Feature Selection** | | **With Feature Selection** | |
|  | **Score** | **UM Score** | **Score** | **UM Score** | **Score** | **UM Score** | **Score** | **UM Score** | **Score** | **UM Score** | **Score** | **UM Score** | **Score** | **UM Score** | **Score** | **UM Score** |
| Accuracy | 0.61897414 | 0.63675094 | 0.61897414 | 0.63675094 | 0.67223482 | 0.70952339 | 0.65985179 | 0.69173496 | 0.64373147 | 0.64546353 | 0.62430108 | 0.64700959 | 0.62218541 | 0.646966 | 0.65616681 | 0.65827376 |
| Misclassification | 0.38102586 | 0.36324906 | 0.38102586 | 0.36324906 | 0.32776518 | 0.29047661 | 0.34014821 | 0.30826504 | 0.35626853 | 0.35453647 | 0.37569892 | 0.35299041 | 0.37781459 | 0.353034 | 0.34383319 | 0.34172624 |
| Sensitivity | 0.66885057 | 0.65172414 | 0.66885057 | 0.65172414 | 0.52218391 | 0.37425287 | 0.5491954 | 0.41563218 | 0.65563218 | 0.68793103 | 0.69344828 | 0.66873563 | 0.68954023 | 0.67954023 | 0.65183908 | 0.65908046 |
| Specificity | 0.60957455 | 0.63380287 | 0.60957455 | 0.63380287 | 0.70041646 | 0.77197861 | 0.6805365 | 0.74323044 | 0.64162175 | 0.63704883 | 0.61156296 | 0.64290381 | 0.60961947 | 0.64103381 | 0.6569982 | 0.65828025 |
| Positive Predictive Value | 0.25081333 | 0.26703683 | 0.25081333 | 0.26703683 | 0.25051264 | 0.25119851 | 0.24396114 | 0.24333019 | 0.28689149 | 0.28190118 | 0.2640922 | 0.27132872 | 0.26507351 | 0.27852996 | 0.27308463 | 0.27785854 |
| Negative Predictive Value | 0.91281258 | 0.91349075 | 0.91281258 | 0.91349075 | 0.88720307 | 0.87037024 | 0.88977987 | 0.87426846 | 0.9124497 | 0.92321922 | 0.91655583 | 0.9131909 | 0.92155464 | 0.92067441 | 0.91319474 | 0.91687551 |
| Positive Likelihood Ratio | 0.00672748 | 0.00655591 | 0.00672748 | 0.00655591 | 0.00525822 | 0.00376962 | 0.00552948 | 0.0041853 | 0.00659654 | 0.00692074 | 0.00697585 | 0.00672972 | 0.00693457 | 0.00683684 | 0.00655988 | 0.00663224 |
| Negative Likelihood Ratio | 174.613136 | 179.523856 | 174.613136 | 179.523856 | 143.367399 | 136.733531 | 146.711782 | 141.659346 | 166.289057 | 176.021875 | 168.735907 | 159.250804 | 173.783965 | 162.216577 | 155.279622 | 156.962734 |
| F1 Score | 0.35676488 | 0.36515086 | 0.35676488 | 0.36515086 | 0.3338439 | 0.28620911 | 0.33713327 | 0.29268984 | 0.37718306 | 0.38664019 | 0.37314308 | 0.37916637 | 0.36482153 | 0.3788994 | 0.37370948 | 0.37766763 |

Abbreviations: UM – upsampled minority.

**eTable 8: Escitalopram performance statistics**

| **Youden index optimized parameters** | **Logistic Regression** | | | | **Decision tree** | | | | **Random Forest** | | | | **XGBoost** | | | |
| --- | --- | --- | --- | --- | --- | --- | --- | --- | --- | --- | --- | --- | --- | --- | --- | --- |
|  | **Without Feature Selection** | | **With Feature Selection** | | **Without Feature Selection** | | **With Feature Selection** | | **Without Feature Selection** | | **With Feature Selection** | | **Without Feature Selection** | | **With Feature Selection** | |
|  | **Score** | **UM Score** | **Score** | **UM Score** | **Score** | **UM Score** | **Score** | **UM Score** | **Score** | **UM Score** | **Score** | **UM Score** | **Score** | **UM Score** | **Score** | **UM Score** |
| Accuracy | 0.59427829 | 0.60426789 | 0.70156222 | 0.60503861 | 0.79237897 | 0.75379121 | 0.76129938 | 0.78326106 | 0.59615979 | 0.65265221 | 0.62183843 | 0.63964508 | 0.69925156 | 0.71322988 | 0.71437927 | 0.78017672 |
| Misclassification | 0.40572171 | 0.39573211 | 0.29843778 | 0.39496139 | 0.20762103 | 0.24620879 | 0.23870062 | 0.21673894 | 0.40384021 | 0.34734779 | 0.37816157 | 0.36035492 | 0.30074844 | 0.28677012 | 0.28562073 | 0.21982328 |
| Sensitivity | 0.6575 | 0.64541667 | 0.55291667 | 0.64541667 | 0.29708333 | 0.39916667 | 0.43375 | 0.28875 | 0.67333333 | 0.61125 | 0.64333333 | 0.62291667 | 0.59208333 | 0.51166667 | 0.53875 | 0.44291667 |
| Specificity | 0.58987621 | 0.60139846 | 0.71121445 | 0.60221813 | 0.8242372 | 0.77666778 | 0.78204082 | 0.81480596 | 0.59118602 | 0.65547675 | 0.62067247 | 0.64075109 | 0.70632988 | 0.72591335 | 0.72564905 | 0.80173804 |
| Positive Predictive Value | 0.09961328 | 0.10331328 | 0.12129376 | 0.10406796 | NA | 0.1206422 | 0.11017661 | 0.09619976 | 0.10452707 | 0.11930395 | 0.10694406 | 0.12463937 | 0.11932547 | 0.1260981 | 0.1215887 | 0.13880832 |
| Negative Predictive Value | 0.96462201 | 0.96551866 | 0.9622135 | 0.965554 | 0.94860692 | 0.95327958 | 0.95670799 | 0.94687859 | 0.966962 | 0.96501353 | 0.96528575 | 0.96570938 | 0.96507986 | 0.95924714 | 0.96241003 | 0.95870269 |
| Positive Likelihood Ratio | 0.00661266 | 0.00649033 | 0.00556674 | 0.00649035 | 0.00299452 | 0.00402029 | 0.0043706 | 0.00290805 | 0.00677097 | 0.00614971 | 0.00647168 | 0.0062658 | 0.00596175 | 0.00515146 | 0.00542475 | 0.0044625 |
| Negative Likelihood Ratio | 180.517987 | 205.627394 | 146.203849 | 205.58167 | 122.087184 | 143.231604 | 128.288007 | 141.031686 | 184.988031 | 166.322324 | 170.01537 | 170.118895 | 143.996892 | 148.097591 | 143.828557 | 128.729479 |
| F1 Score | 0.17070519 | 0.17354854 | 0.19157887 | 0.17436264 | NA | 0.17491421 | 0.17250145 | NA | 0.17564638 | 0.18817168 | 0.17862169 | 0.19073641 | 0.19489012 | 0.19175178 | 0.19230064 | 0.19368879 |

Abbreviations: UM – upsampled minority.

**eTable 9: Citalopram performance statistics**

| **Youden index optimized parameters** | **Logistic Regression** | | | | **Decision tree** | | | | **Random Forest** | | | | **XGBoost** | | | |
| --- | --- | --- | --- | --- | --- | --- | --- | --- | --- | --- | --- | --- | --- | --- | --- | --- |
|  | **Without Feature Selection** | | **With Feature Selection** | | **Without Feature Selection** | | **With Feature Selection** | | **Without Feature Selection** | | **With Feature Selection** | | **Without Feature Selection** | | **With Feature Selection** | |
|  | **Score** | **UM Score** | **Score** | **UM Score** | **Score** | **UM Score** | **Score** | **UM Score** | **Score** | **UM Score** | **Score** | **UM Score** | **Score** | **UM Score** | **Score** | **UM Score** |
| Accuracy | 0.63573136 | 0.70320374 | 0.70479807 | 0.66491461 | 0.81625477 | 0.77812839 | 0.79685453 | 0.79558469 | 0.66775367 | 0.67916918 | 0.64429677 | 0.66708459 | 0.68739401 | 0.65537673 | 0.62615029 | 0.71467953 |
| Misclassification | 0.36426864 | 0.29679626 | 0.29520193 | 0.33508539 | 0.18374523 | 0.22187161 | 0.20314547 | 0.20441531 | 0.33224633 | 0.32083082 | 0.35570323 | 0.33291541 | 0.31260599 | 0.34462327 | 0.37384971 | 0.28532047 |
| Sensitivity | 0.65394089 | 0.59211823 | 0.57832512 | 0.62573892 | 0.36637931 | 0.36711823 | 0.36280788 | 0.33165025 | 0.67820197 | 0.63682266 | 0.67450739 | 0.6408867 | 0.61884236 | 0.62549261 | 0.66847291 | 0.5520936 |
| Specificity | 0.63396116 | 0.71450744 | 0.71768963 | 0.66883726 | 0.86105602 | 0.81910528 | 0.84001852 | 0.84176433 | 0.6666821 | 0.68347993 | 0.64126361 | 0.66993129 | 0.69426915 | 0.65828074 | 0.62209376 | 0.73087157 |
| Positive Predictive Value | 0.17518735 | 0.20530218 | 0.18750373 | 0.16725848 | 0.21094745 | 0.18340272 | NA | NA | 0.17451057 | 0.17282444 | 0.1637573 | 0.17892627 | 0.17086796 | 0.16172844 | 0.15622341 | 0.17982429 |
| Negative Predictive Value | 0.9521268 | 0.94860244 | 0.94606389 | 0.94856909 | 0.93225896 | 0.9312551 | 0.93067202 | 0.92952979 | 0.95541932 | 0.95057617 | 0.95383298 | 0.95184118 | 0.9482123 | 0.94690727 | 0.95138459 | 0.9433858 |
| Positive Likelihood Ratio | 0.00657751 | 0.00596158 | 0.0058234 | 0.006298 | 0.00369495 | 0.00369795 | 0.00365684 | 0.00334082 | 0.00682645 | 0.00641117 | 0.00678716 | 0.00644978 | 0.00623145 | 0.00629544 | 0.00672513 | 0.00556047 |
| Negative Likelihood Ratio | 173.781047 | 147.157869 | 142.248538 | 153.672945 | 116.148515 | 131.510481 | 123.093122 | 129.005195 | 152.843643 | 149.239829 | 160.730195 | 158.619105 | 144.193436 | 155.020458 | 168.010602 | 138.370373 |
| F1 Score | 0.25860784 | 0.27369829 | 0.26608794 | 0.25696604 | 0.25471735 | NA | NA | NA | 0.27420566 | 0.26892522 | 0.25952295 | 0.26851814 | 0.26647179 | 0.25306236 | 0.24980242 | 0.26350084 |

Abbreviations: UM – upsampled minority.

**eTable 10: Fluoxetine performance statistics**

| **Youden index optimized parameters** | **Logistic Regression** | | | | **Decision tree** | | | | **Random Forest** | | | | **XGBoost** | | | |
| --- | --- | --- | --- | --- | --- | --- | --- | --- | --- | --- | --- | --- | --- | --- | --- | --- |
|  | **Without Feature Selection** | | **With Feature Selection** | | **Without Feature Selection** | | **With Feature Selection** | | **Without Feature Selection** | | **With Feature Selection** | | **Without Feature Selection** | | **With Feature Selection** | |
|  | **Score** | **UM Score** | **Score** | **UM Score** | **Score** | **UM Score** | **Score** | **UM Score** | **Score** | **UM Score** | **Score** | **UM Score** | **Score** | **UM Score** | **Score** | **UM Score** |
| Accuracy | 0.68560886 | 0.67898714 | 0.6922265 | 0.67898714 | 0.81355682 | 0.7377659 | 0.80394915 | 0.84628283 | 0.68116589 | 0.69326704 | 0.70395187 | 0.64363062 | 0.71164804 | 0.6521489 | 0.70765954 | 0.69036656 |
| Misclassification | 0.31439114 | 0.32101286 | 0.3077735 | 0.32101286 | 0.18644318 | 0.2622341 | 0.19605085 | 0.15371717 | 0.31883411 | 0.30673296 | 0.29604813 | 0.35636938 | 0.28835196 | 0.3478511 | 0.29234046 | 0.30963344 |
| Sensitivity | 0.5770751 | 0.58003953 | 0.56837945 | 0.58003953 | 0.36778656 | 0.44288538 | 0.28379447 | 0.24841897 | 0.58478261 | 0.57075099 | 0.57134387 | 0.60335968 | 0.51126482 | 0.62371542 | 0.56304348 | 0.59367589 |
| Specificity | 0.69576546 | 0.688 | 0.7038008 | 0.688 | 0.85401124 | 0.76448835 | 0.85121767 | 0.90059438 | 0.6900257 | 0.70435341 | 0.71602249 | 0.64753574 | 0.73019116 | 0.65467791 | 0.72089317 | 0.6992498 |
| Positive Predictive Value | 0.17983441 | 0.18589133 | 0.18235206 | 0.18589133 | 0.19510778 | 0.14974647 | 0.15607363 | 0.22684869 | 0.15369219 | 0.15513231 | 0.15714541 | 0.13950927 | 0.15548057 | 0.15720725 | 0.16140787 | 0.15978767 |
| Negative Predictive Value | 0.94945273 | 0.94873541 | 0.94887664 | 0.94873541 | 0.93774886 | 0.93901861 | 0.92951716 | 0.92976496 | 0.94979399 | 0.94905917 | 0.94977396 | 0.94880692 | 0.94421593 | 0.95150496 | 0.94948434 | 0.95172795 |
| Positive Likelihood Ratio | 0.00580837 | 0.00583846 | 0.00572134 | 0.00583846 | 0.00370858 | 0.00446128 | 0.00286157 | 0.00250625 | 0.00588675 | 0.00574667 | 0.00575351 | 0.00607124 | 0.00514824 | 0.00627608 | 0.00566986 | 0.00597678 |
| Negative Likelihood Ratio | 153.948274 | 152.100843 | 152.406456 | 152.100843 | 117.493339 | 134.12897 | 117.955924 | 111.543604 | 148.494146 | 143.580728 | 141.280369 | 161.310628 | 142.758483 | 163.634119 | 140.845675 | 147.246298 |
| F1 Score | 0.24952397 | 0.2470001 | 0.25104809 | 0.2470001 | 0.2400224 | 0.21406109 | 0.18701845 | 0.21731662 | 0.23645964 | 0.23658988 | 0.24317382 | 0.22274123 | 0.23053083 | 0.2416635 | 0.24471591 | 0.24595124 |

Abbreviations: UM – upsampled minority.

**eTable 11: Sertraline performance statistics**

| **Youden index optimized parameters** | **Logistic Regression** | | | | **Decision tree** | | | | **Random Forest** | | | | **XGBoost** | | | |
| --- | --- | --- | --- | --- | --- | --- | --- | --- | --- | --- | --- | --- | --- | --- | --- | --- |
|  | **Without Feature Selection** | | **With Feature Selection** | | **Without Feature Selection** | | **With Feature Selection** | | **Without Feature Selection** | | **With Feature Selection** | | **Without Feature Selection** | | **With Feature Selection** | |
|  | **Score** | **UM Score** | **Score** | **UM Score** | **Score** | **UM Score** | **Score** | **UM Score** | **Score** | **UM Score** | **Score** | **UM Score** | **Score** | **UM Score** | **Score** | **UM Score** |
| Accuracy | 0.62954996 | 0.50793894 | 0.62905613 | 0.50793894 | 0.76280788 | 0.78230858 | 0.7583829 | 0.68699994 | 0.62337895 | 0.54696163 | 0.59253847 | 0.63055039 | 0.60217418 | 0.5321237 | 0.59879462 | 0.56616919 |
| Misclassification | 0.37045004 | 0.49206106 | 0.37094387 | 0.49206106 | 0.23719212 | 0.21769142 | 0.2416171 | 0.31300006 | 0.37662105 | 0.45303837 | 0.40746153 | 0.36944961 | 0.39782582 | 0.4678763 | 0.40120538 | 0.43383081 |
| Sensitivity | 0.57664884 | 0.70588235 | 0.57664884 | 0.70588235 | 0.36987522 | 0.27914439 | 0.32807487 | 0.44108734 | 0.66836007 | 0.74349376 | 0.69901961 | 0.64919786 | 0.67531194 | 0.73841355 | 0.67130125 | 0.7026738 |
| Specificity | 0.63422746 | 0.49017477 | 0.63368837 | 0.49017477 | 0.79849216 | 0.82769904 | 0.79740457 | 0.70938759 | 0.61909182 | 0.5290924 | 0.58284135 | 0.62891415 | 0.59550039 | 0.51354665 | 0.59202388 | 0.55389966 |
| Positive Predictive Value | 0.132787 | 0.11688078 | 0.13268426 | 0.11688078 | 0.15833507 | 0.16523094 | 0.13780437 | 0.14364446 | 0.14092893 | 0.14165513 | 0.13575093 | 0.14655974 | 0.13440649 | 0.12877629 | 0.1343557 | 0.13500561 |
| Negative Predictive Value | 0.94557336 | 0.95225527 | 0.94552051 | 0.95225527 | 0.93358472 | 0.9274602 | 0.93037275 | 0.93632405 | 0.95683034 | 0.96154549 | 0.95735598 | 0.95437679 | 0.95396834 | 0.95833914 | 0.95516701 | 0.95473329 |
| Positive Likelihood Ratio | 0.00580058 | 0.00709066 | 0.00580054 | 0.00709066 | 0.00372695 | 0.00281348 | 0.0033047 | 0.00443762 | 0.0067235 | 0.0074711 | 0.00702994 | 0.00653069 | 0.00679261 | 0.00741984 | 0.00675024 | 0.00706374 |
| Negative Likelihood Ratio | 169.588276 | 236.899913 | 169.925818 | 236.899913 | 131.234364 | 122.232239 | 132.392094 | 155.564794 | 171.613679 | 217.715845 | 181.846866 | 171.506474 | 174.2427 | 233.823349 | 184.06699 | 201.990327 |
| F1 Score | 0.20881739 | 0.19661165 | 0.20866239 | 0.19661165 | 0.21333663 | 0.16616695 | 0.18093817 | 0.18778814 | 0.23010495 | 0.22627787 | 0.22550071 | 0.23254703 | 0.22254747 | 0.21515577 | 0.21868763 | 0.22117457 |

Abbreviations: UM – upsampled minority.

**eTable 12: Paroxetine performance statistics**

| **Youden index optimized parameters** | **Logistic Regression** | | | | **Decision tree** | | | | **Random Forest** | | | | **XGBoost** | | | |
| --- | --- | --- | --- | --- | --- | --- | --- | --- | --- | --- | --- | --- | --- | --- | --- | --- |
|  | **Without Feature Selection** | | **With Feature Selection** | | **Without Feature Selection** | | **With Feature Selection** | | **Without Feature Selection** | | **With Feature Selection** | | **Without Feature Selection** | | **With Feature Selection** | |
|  | **Score** | **UM Score** | **Score** | **UM Score** | **Score** | **UM Score** | **Score** | **UM Score** | **Score** | **UM Score** | **Score** | **UM Score** | **Score** | **UM Score** | **Score** | **UM Score** |
| Accuracy | 0.59636364 | 0.67727273 | 0.59545455 | 0.67727273 | 0.91181818 | 0.91181818 | 0.83363636 | 0.84818182 | 0.62818182 | 0.63727273 | 0.67363636 | 0.65454545 | 0.62272727 | 0.62636364 | 0.61909091 | 0.67272727 |
| Misclassification | 0.40363636 | 0.32272727 | 0.40454545 | 0.32272727 | 0.08818182 | 0.08818182 | 0.16636364 | 0.15181818 | 0.37181818 | 0.36272727 | 0.32636364 | 0.34545455 | 0.37727273 | 0.37363636 | 0.38090909 | 0.32727273 |
| Sensitivity | 0.67 | 0.53777778 | 0.67 | 0.53777778 | 0 | 0 | 0.29666667 | 0.15222222 | 0.58888889 | 0.56 | 0.57111111 | 0.52888889 | 0.58111111 | 0.52666667 | 0.59888889 | 0.45555556 |
| Specificity | 0.58914851 | 0.69126733 | 0.58815842 | 0.69126733 | 1 | 1 | 0.88520792 | 0.91524752 | 0.63220792 | 0.64552475 | 0.68427723 | 0.66750495 | 0.62759406 | 0.63652475 | 0.62139604 | 0.69436634 |
| Positive Predictive Value | 0.14709276 | 0.16563708 | 0.14683766 | 0.16563708 | NA | NA | 0.3008622 | NA | 0.15278495 | 0.14427706 | 0.17755012 | 0.15692539 | 0.14874779 | 0.13457019 | 0.16492906 | 0.14006162 |
| Negative Predictive Value | 0.94970943 | 0.94239518 | 0.94963011 | 0.94239518 | 0.91181818 | 0.91181818 | 0.92950686 | 0.91922873 | 0.94279163 | 0.93714477 | 0.94710708 | 0.92981505 | 0.94086487 | 0.90854108 | 0.94254865 | 0.92831134 |
| Positive Likelihood Ratio | 0.00673732 | 0.00541194 | 0.00673726 | 0.00541194 | 0 | 0 | 0.00299211 | 0.00153454 | 0.00592248 | 0.00563372 | 0.00574616 | 0.00531996 | 0.00584431 | 0.00529659 | 0.00602218 | 0.00458499 |
| Negative Likelihood Ratio | 201.502618 | 152.752769 | 201.7958 | 152.752769 | 100 | 100 | 113.45369 | 110.212076 | 194.462627 | 182.551152 | 161.920971 | 293.714478 | 196.126914 | 631.950982 | 206.000687 | 162.124785 |
| F1 Score | 0.23481445 | 0.22871855 | 0.23445147 | 0.22871855 | NA | NA | 0.23036899 | NA | 0.23184678 | 0.21991546 | 0.235204 | 0.22508342 | 0.2240315 | 0.2074761 | 0.2374106 | 0.20339301 |

Abbreviations: UM – upsampled minority.

**eTable 13: Combined SSRI performance statistics**

| **Youden index optimized parameters** | **Logistic Regression** | | | | **Decision tree** | | | | **Random Forest** | | | | **XGBoost** | | | |
| --- | --- | --- | --- | --- | --- | --- | --- | --- | --- | --- | --- | --- | --- | --- | --- | --- |
|  | **Without Feature Selection** | | **With Feature Selection** | | **Without Feature Selection** | | **With Feature Selection** | | **Without Feature Selection** | | **With Feature Selection** | | **Without Feature Selection** | | **With Feature Selection** | |
|  | **Score** | **UM Score** | **Score** | **UM Score** | **Score** | **UM Score** | **Score** | **UM Score** | **Score** | **UM Score** | **Score** | **UM Score** | **Score** | **UM Score** | **Score** | **UM Score** |
| Accuracy | 0.64676255 | 0.62768177 | 0.64591099 | 0.62768177 | 0.74430409 | 0.79955579 | 0.74487172 | 0.79955579 | 0.66754099 | 0.64058701 | 0.65491909 | 0.68357645 | 0.69740644 | 0.64292305 | 0.64123733 | 0.72790914 |
| Misclassification | 0.35323745 | 0.37231823 | 0.35408901 | 0.37231823 | 0.25569591 | 0.20044421 | 0.25512828 | 0.20044421 | 0.33245901 | 0.35941299 | 0.34508091 | 0.31642355 | 0.30259356 | 0.35707695 | 0.35876267 | 0.27209086 |
| Sensitivity | 0.62489899 | 0.64690909 | 0.62590909 | 0.64690909 | 0.45553535 | 0.35628283 | 0.45553535 | 0.35628283 | 0.61693939 | 0.6390404 | 0.63686869 | 0.58965657 | 0.57852525 | 0.63590909 | 0.64681818 | 0.53661616 |
| Specificity | 0.64847305 | 0.62625576 | 0.64748033 | 0.62625576 | 0.76620405 | 0.83322468 | 0.76681468 | 0.83322468 | 0.67144869 | 0.64076627 | 0.65633128 | 0.69075212 | 0.70646374 | 0.64349847 | 0.64083533 | 0.74250913 |
| Positive Predictive Value | 0.12354642 | 0.12062359 | 0.12304269 | 0.12062359 | 0.1610822 | 0.16082221 | 0.16178037 | 0.16082221 | 0.13184233 | 0.12772712 | 0.13432511 | 0.13689732 | 0.1396773 | 0.12795764 | 0.1282718 | 0.15114135 |
| Negative Predictive Value | 0.95870662 | 0.95948875 | 0.9587507 | 0.95948875 | 0.95066743 | 0.94538083 | 0.95069788 | 0.94538083 | 0.95966167 | 0.96011357 | 0.96101776 | 0.95727371 | 0.95698513 | 0.96107627 | 0.96021742 | 0.95557439 |
| Positive Likelihood Ratio | 0.00628868 | 0.00650891 | 0.00629882 | 0.00650891 | 0.00458633 | 0.00359028 | 0.00458636 | 0.00359028 | 0.0062096 | 0.00642997 | 0.006409 | 0.00593651 | 0.00582547 | 0.00639791 | 0.00650894 | 0.00540479 |
| Negative Likelihood Ratio | 157.662455 | 163.561856 | 157.697557 | 163.561856 | 151.158287 | 128.851495 | 151.07611 | 128.851495 | 153.638078 | 161.846686 | 158.360995 | 148.173 | 144.139164 | 168.13265 | 160.090226 | 138.366627 |
| F1 Score | 0.20371343 | 0.2012388 | 0.20322138 | 0.2012388 | 0.22275487 | 0.20693763 | 0.22343784 | 0.20693763 | 0.21321497 | 0.20783903 | 0.21582988 | 0.21708741 | 0.22058788 | 0.20785641 | 0.21059315 | 0.2281367 |

Abbreviations: UM – upsampled minority.
